# Mechanochemical Extrusion for Sustainable Synthesis of Amides and Amines via the Leuckart Reaction

**DOI:** 10.1021/acssuschemeng.5c06980

**Published:** 2025-10-07

**Authors:** Francesco Zorzetto, Alvise Perosa, Daily Rodriguez-Padron, Maurizio Selva

**Affiliations:** Department of Molecular Science and Nanosystems, 19047 Ca’ Foscari University of Venice, Via Torino 155, Mestre, Venezia 30175, Italy

**Keywords:** mechanochemistry, solvent-free, extrusion, reductive amination, heterogeneous catalysis

## Abstract

An extrusion-based, solvent-free method was developed for the synthesis of amides and amines via the Leuckart reaction, representing the first adaptation of this classical transformation into a continuous mechanochemical platform. The production of amides was demonstrated through the model reaction between ammonium formate and vanillin, leading to vanillyl formamide, while the synthesis of tertiary amines was investigated using ammonium formate, vanillin, and morpholine as reactants. A thorough parametric analysis revealed that the mechanochemical-based strategy allowed extremely fast processes: at 100–150 °C, reactions were quantitative in 5–15 min with product selectivity >99% for vanillin formamide and up to 83% for the corresponding tertiary amine. An investigation of the substrate scope (four examples) confirmed the robustness of the protocol for both reaction types. Benzyl-type amides and tertiary amines were synthesized with high conversion and selectivity, exceeding 95%, across different aldehydes. Moreover, morpholine could be successfully replaced by other secondary amines (diethanolamine and *N*-methyl-*p*-anisidine), thereby further demonstrating the versatility of the reactive extrusion for the preparation of amines. Overall, this work underscores the novelty and impact of solvent-free continuous extrusion, establishing it as a scalable and sustainable alternative to liquid-phase synthesis, and marking a significant step forward in green chemistry practices.

## Graphical abstract

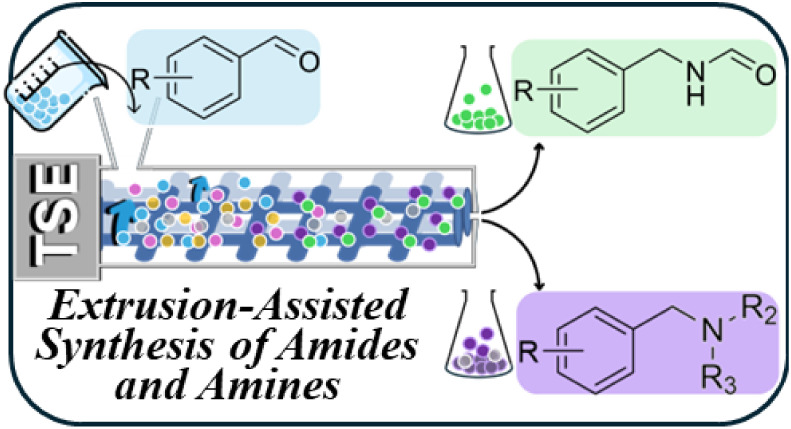

## Introduction

Mechanochemistry—recognized by the IUPAC as one of the top ten technologies poised to change the chemistry world—offers excellent options for the design of processes aligned with the principles of green chemistry.^1,2^

Indeed, the exploitation of mechanical energy to drive chemical reactions, typically through ball milling or extrusion, not only eliminates the need of bulk solvents, but very often, improves kinetics and selectivity,^3,4^ thereby achieving conditions suitable to more sustainable and scalable processes. Among other applications, mechanochemistry is also becoming progressively popular in the field of pharmaceuticals and agrochemicals, particularly for the formation of carbon–nitrogen (C–N) bonds in the synthesis of intermediates such as amides and amines. One of the most attractive and widely applied approaches for C–N bond formation is the reductive amination reaction, which predominantly affords amines and accounts for at least a quarter of such transformations.^5,6^

### Amide Synthesis

Amides are commonly prepared through acyl substitution reactions involving carboxylic acid derivatives or coupling agents. In this sense, mechanochemical-assisted protocols have been mainly described under ball milling conditions (Figure 1, top left). A notable example reported the conversion of esters to primary amides using calcium nitride. This method was compatible with diverse functional groups and allowed the retention of stereochemical integrity, as demonstrated by the synthesis of *N*-Boc (*tert*-butyloxycarbonyl) dipeptide esters and rufinamide (Figure 1a).^7^ Another procedure was designed through enzymatic mechanochemistry using papain to catalyze the formation of peptides from amino acid esters and amine hydrochlorides.^8^ The biocatalyst’s stability was ensured, and the synthesis of α,α- and α,β-dipeptides was achieved. In addition, a significant step forward was the mechanochemical-assisted coupling of carboxylic groups with amines in the presence of either carbonyl diimidazole (CDI)^9^ or 2,4,6-trichloro-1,3,5-triazine and catalytic PPh_3_ (Figure 1b,c).^10^ The latter protocol was suitable even for aromatic, aliphatic, and *N*-protected amino acids, offering compatibility with Fmoc (Fluorenylmethyloxycarbonyl), Cbz (Benzyloxycarbonyl), and Boc groups. Yields up to 96% were achieved with minimal environmental impact.^10^

**Figure 1:**
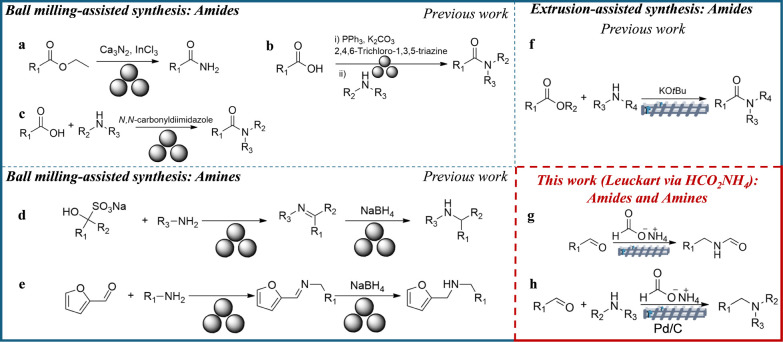
Mechanochemical-assisted synthesis of amides and amines. Examples **a–f**: selected literature methods; Examples **g,h**: Extrusion-based Leuckart process was explored in this work.

### Amine Synthesis

Like in the case of amides, the mechanochemical synthesis of amines has been mostly designed via ball mill procedures. In this field, a metal-catalyzed oxidative C­(sp^3^)–H amination through nitrene transfer was developed by employing Rh_2_(esp)_2_ as a catalyst and C–H bonds from a variety of substrates including amides, amines, amino acids, and even hydrocarbons as cycloalkanes. The reaction took advantage of mechanochemical conditions to increase catalyst stability, reduce catalyst loading, and shorten reaction times, thereby improving both the efficiency and sustainability of the process.^11^ Another innovative strategy was devised through the catalytic transfer hydrogenation (CTH) of aromatic nitro compounds to produce substituted anilines. In the presence of ammonium formate as a hydrogen source, this process enabled the synthesis of pharmaceutical intermediates like procainamide and paracetamol.^12^

Recent studies explored ball milling conditions to induce reductive amination processes. For example, the use of Bertagnini’s salts (aldehyde-bisulfite adducts) as solid surrogates for aldehydes and ketones was described to prepare either benzimidazoles or secondary aromatic amines (Figure 1d),^13^ while the reductive amination of chitosan and furfural was achieved via liquid-assisted grinding (LAG) (Figure 1e).^14^

### Novel Approach

The methods described above are based on the most common techniques of milling/grinding to achieve mechanochemical transformations. However, especially during milling, the lack of heat control is a significant drawback that may induce side reactions.^15^ A significant advancement in this regard was reported in a recent study, where the mechanochemical synthesis of amides from esters was described by the translation of a small-scale ball-milled reaction into a continuous reactive extrusion process (Figure 1f). This work offered a practical, solvent-free, and cost-effective continuous-flow approach for amide synthesis, demonstrating the scalability of the reaction: not only the amidation was successful for 36 amides in a variety of physical form combinations (liquid–liquid, solid–liquid, and solid–solid), but after 7 h of extrusion at 50–100 °C, up to 500 g of the desired products were obtained.^16^

Based on these considerations, an innovative extrusion-based method was developed herein to synthesize amides and amines using a solvent-free Leuckart-type reaction (Figure 1g,h).

The Leuckart reaction is traditionally carried out using liquid formamide or formic acid for the reductive amination of carbonyl compounds. Albeit extensively studied for the synthesis of a variety of primary, secondary, and tertiary amines,^17–20^ the batch reaction in solution is characterized by harsh conditions, particularly the combined need of high temperatures (160–185 °C) and long reaction times (6 to 25 h). Not to mention the use of excess reactant(s) brings about low atom efficiency.

An alternative version of the Leuckart protocol has been designed with ammonium formate acting both as the nitrogen source and as a reducing agent. Under the reaction conditions, the most accepted mechanism proposes the dissociation of ammonium formate into formic acid and ammonia, which in turn converts a carbonyl compound into an amine through subsequent steps of nucleophilic addition, dehydration, and decarboxylation/reduction (step 1, Scheme 1; further details are reported in Scheme S1). Interestingly, if an excess ammonium formate is used, the amine product reacts further through a formylation reaction producing the corresponding formamide derivative (step 2, Scheme 1).^21,22^

**Scheme 1:**
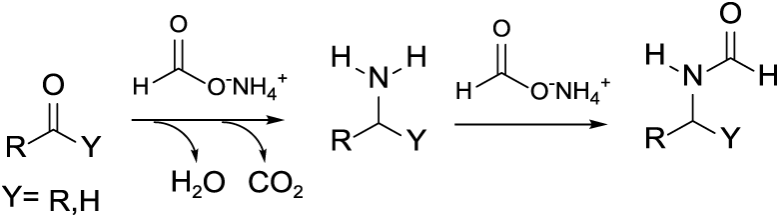
Leuckart Reaction with Ammonium Formate

Alternatively, even the direct condensation of formamide originated by the thermal treatment of ammonium formate, to a carbonyl has been suggested as the initial step.^23^

In this study, taking advantage of the solid nature of ammonium formate, the Leuckart protocol was revisited with the aim of designing and optimizing it under mechanochemical conditions, particularly via continuous reactive extrusion.

The analysis of model reactions of ammonium formate with vanillin or a mixture of vanillin and morpholine demonstrated the potential of mechanochemistry as a streamlined approach for the synthesis of both formamides and amines, respectively. These results along with further investigations on the substrate scope highlighted how reactive extrusion enabled the conversion of the conventional liquid-based Leuckart reaction into a greener and far more efficient procedure by which an excellent improvement of the kinetics was achieved. Indeed, the extrusion-assisted process allowed a continuous-flow synthesis of amide products in reaction times as short as 5 min and the production of tertiary amines without supplying extra hydrogenation agents and with minimal, if any, use of solvents. The method proved to be successful also for multigram preparations, thereby offering prospects for further scalable applications.

## Experimental Section

All chemicals employed during the reactions were commercially available compounds sourced from Sigma-Aldrich and employed without further purification. Albeit all chemicals were ACS grade, some of them displayed purities <99%: these included vanillin (97%), ammonium formate (97%), 2-hydroxy-5-methylbenzaldehyde (98%), chlorobenzaldehyde (98%), and *N*-methyl-*p*-anisidine (95%).

All reactions, either carried out in the extruder (as described below) or under batch conditions (as described in the results and discussion section), were performed in duplicate to verify reproducibility. The structure of the products was assigned by both mass and NMR spectrometry. NMR spectra were recorded by a Bruker Avance III HD 400 WB. The chemical shifts were reported downfield from tetramethylsilane (TMS), and CDCl_3_ was used as the solvent.

Mass spectra were recorded by GC-MS using an Agilent 7820A GC/5977B equipped with an HP5-MS capillary column (*L* = 30 m, Ø = 0.32 mm, film = 0.25 mm) and coupled to a High Efficiency Source (HES) MSD; (ii) LC-MS using an Agilent 1260 Infinity coupled to single quad Mass Spectrometer (LC/MSD) G6125B. GC (flame ionization detector; FID) analyses were performed with an Elite-624 capillary column (*L* = 30 m, Ø = 0.32 mm, and film = 1.8 mm).

## Mechanochemical Experiments

### Amide Synthesis via an Extrusion-Based Leuckart-Type Approach

A two-screen ZE 12 HMI extruder from Three Tec was used for all of the reactive extrusion tests (Figure 2). In a typical reaction for the synthesis of amides, a mixture of vanillin (**1a**, 5 mmol) and ammonium formate (3 equiv with respect to the aldehyde) was introduced into the extruder and set to react at 150 °C. The rotation speed of the screw was set to 100 rpm. At the outlet of the extrusion barrel, a sample of the mixture (5–10 mg) was dissolved in methanol (0.5 mL) and analyzed by GC/FID to evaluate both the conversion of the aldehyde and the selectivity toward the corresponding amide. The structures of the main product and other side derivatives were determined by GC-MS, LC-MS, and NMR (Figures S1–S34). A variety of experiments were carried out to explore the effect of the mass loading (5 to 25 mmol of vanillin), the temperature (130–180 °C), the reaction time (5–15 min), and the rotation speed of the extruder screw (50–100 rpm). Details are given in the Results and Discussion section. Moreover, the extrusion-based procedure was run using other four solid aldehydes (5 mmol each) such as *ortho*-vanillin (**1b**), 5-methylsalicylaldehyde (**1c**), 4-chlorobenzaldehyde (**1d**) and 4-bromobenzaldehyde (**1e**), which were set to react at 150 °C for 15 min (conditions of Table 2). In this case, the screw rotation speed was 50 rpm, and the ammonium formate:aldehyde molar ratio (*Q*) was 3.

**Figure 2:**
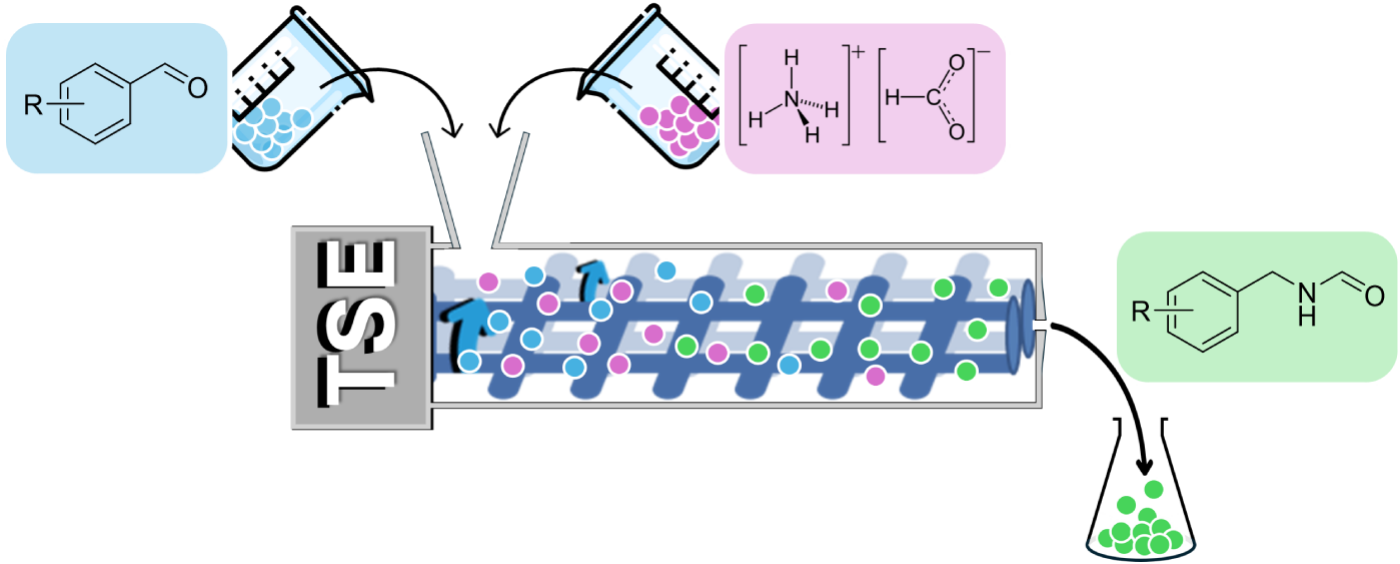
Schematic representation of the extrusion-assisted approach used in this study for the Leuckart reaction. Reactions were carried out using vanillin and ammonium formate as model substrates. TSE: Twin Screw Extruder.

### Amine Synthesis via an Extrusion-Based Leuckart-Type Approach

A two-screen ZE 12 HMI extruder from Three Tec was used for all of the reactive extrusion tests (Figure 5). In a typical reaction for the synthesis of amines, an equimolar mixture of vanillin and morpholine (5 mmol each), ammonium formate (3 equiv with respect to the aldehyde), and Pd/C (100 mg) was introduced into the extruder and set to react at 100 °C. The rotation speed of the screw was set to 50 rpm. The conversion and selectivity and the structures of the products were determined as described above for the synthesis of amides.

The extrusion-based procedure was run using other aldehydes (5 mmol each) such as *ortho*-vanillin (**1b**), 5-methylsalicylaldehyde (**1c**), and other secondary amines including diethanolamine and *N*-methyl-*p*-anisidine (conditions of Table 3). Also, primary amines, both aromatic and aliphatic species, as anisidine, *p*-aminophenol, and decylamine were considered: their use, however, proved unsuccessful toward the preparation of the corresponding secondary amines.

## Results and Discussion

### Extrusion-Based Synthesis of Amides via the Leuckart Reaction

The mechanochemically assisted Leuckart reaction was initially investigated using vanillin (**1a**) as a substrate. Vanillin served as a benchmark reagent not only for its solid nature suitable for mechanochemistry, but for it is an archetypal compound for sustainable syntheses being the only phenolic manufactured on an industrial scale from biomass.^24^

In the context of the Leuckart reaction, it is reasonable to assume that the mechanochemical environment can alter key aspects of the classical solution-based pathway. In particular, the absence of solvent and the intimate contact between reactants can increase the local concentration and collision frequency. Mechanical energy also generates transient regions of high temperature and pressure, lowering activation barriers and accelerating iminium ion formation and reduction. In addition, the continuous removal of water as a byproduct helps shift the equilibrium toward the desired products. These features illustrate how solid-state conditions can modulate and accelerate classical transformations.

The experimental setup developed for this work made use of a mini-twin screw extruder as schematized in Figure 2.

#### Parametric Analysis

The study of the Leuckart process via reactive extrusion had no precedent in the literature. A parametric analysis of the reaction was therefore carried out by selecting conditions based on previous studies reporting the reductive amination of carbonyl compounds with ammonium formate in the conventional batch mode.^21^ In particular, the temperature and the ammonium formate:vanillin molar ratio (*Q*) were varied from 130 to 180 °C, and from 1 to 3, respectively.

The reaction time was ranged from 1 to 15 min and the screw rotation speed of the extruder was set from 50 to 100 rpm. All reactions were performed using 5 mmol of vanillin. At the end of each experiment, the reaction mixture was analyzed by NMR, GC/MS, and LC-MS techniques. Two products were observed: (i) vanillyl formamide (**2a**) which was the expected derivative of the reductive amination of vanillin, and (ii) *N*,*N*-bis­(4-hydroxy-3-methoxybenzyl)­formamide (**3a**) (Scheme 2; structural details are in the Supporting Information).

**Scheme 2:**
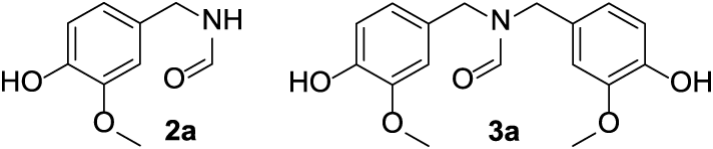
Structures of the Two Products Obtained via the Extrusion-Assisted Leuckart Reaction using Vanillin and Ammonium Formate as Substrates: Vanillin Formamide (**2a**) and *N*,*N*-bis­(4-hydroxy-3-methoxybenzyl) Formamide (**3a**)

The most representative results of the parametric analysis are shown in Figure 3 that display the effect of the reactants’ molar ratio and the screw rotation speed on both the conversion of vanillin and the selectivity toward **2a**. In this work, albeit the Leuckart reaction involved different transformations (Scheme 1), the selectivity was defined according to the following expression:

Si(%)=(ni)/(n0,Vanillin×conv.Vanillin)×100

where Si was the selectivity (%) for compound **i** (**i** = **2a** or **3a**), mol **i** were the total moles of compound **i** (determined by GC calibration), *n*_0,Vanillin_ is the initial number of moles of vanillin, and conv. vanillin is the vanillin conversion (expressed as a fraction).

**Figure 3:**
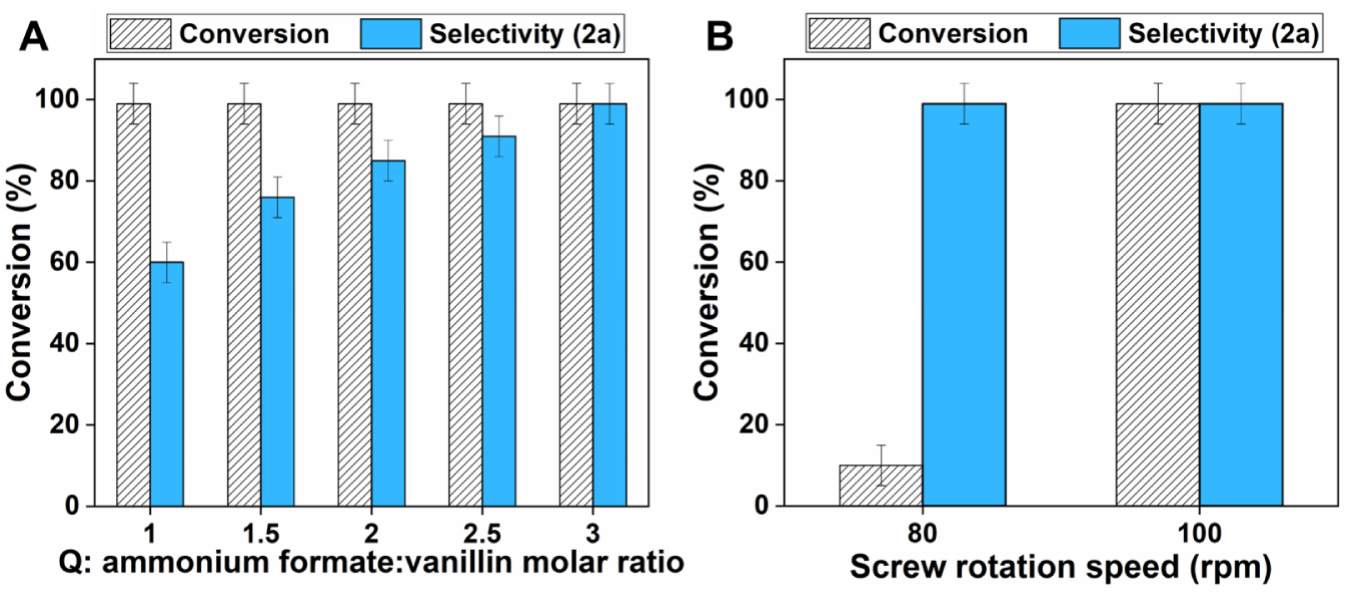
Conversion of vanillin and selectivity toward amide **2a** during the reductive amination with ammonium formate via reactive extrusion. A) Effect of the ammonium formate/vanillin molar ratio (*Q* varied from 1 to 3). Other conditions: 150 °C, 15 min, 50 rpm. B) Effect of the screw speed (revolutions per minute varied from 80 to 100). Other conditions: 150 °C, *Q* = 3, 5 min.

At 150 °C and *Q* = 1, the reaction was quantitative after only 15 min (50 rpm), but the **2a** selectivity did not exceed 60% because of the formation of compound **3a** (Figure 3A). However, as the amount of ammonium formate increased (*Q* from 1 to 3), the undesired derivative **3a** progressively decreased until it completely disappeared and the amide **2a** was achieved as the exclusive reaction product.

Results were consistent with the hypothesis that **3a** was obtained from the condensation of vanillylamine (an intermediate of the synthesis of **2a**, see Scheme S1 for mechanistic details) with vanillin. The higher the *Q* ratio, the lower the availability of unreacted vanillin in the reaction environment, and the lower the formation of the side product **3a**.

Experiments of Figure 3A proved that not only was the reactive extrusion suitable for reductive amination, but more strikingly, an extremely fast process took place by which the conversion of vanillin was completed in a few minutes. This was ascribed to the combined effects of high shear forces, high temperature, and the absence of any solvent in the extruder, which simultaneously acted to improve the contact of the reactants.^25^ Interestingly, in line with these considerations, the parametric analysis demonstrated that the kinetics were further enhanced by increasing the speed at which the screw of the extruder rotated (Figure 3B). At 150 °C, vanillin was recovered unreacted from the extruder after 5 min at 50 rpm (data not shown in the figure). However, if the supply of mechanical energy to the reaction mixture was increased by a higher rotation speed of 80 and 100 rpm, then the conversion of vanillin improved from 10% to 100%, respectively, without any effect on the **2a** selectivity. In summary, the trends reported in Figure 3 were coherent with mechanochemical principles: (i) at the highest screw speed (100 rpm) investigated here, shear forces accelerated the reaction to the level that full conversion was achieved within 5 min; (ii) at such a low residence time, however, decreasing the rotation speed to 80 rpm made the (mechanical) energy input not enough to complete the reaction. Indeed, this proceeded with a poor conversion, slightly exceeding 10%; (iii) finally, at the lowest speed of 50 rpm, no reaction occurred. The process became quantitative only by tripling the reaction time from 5 to 15 min.

The temperature also affected the reaction progress. Under the conditions of Figure 3 (*Q* = 3), a test carried out at 130 °C led to a negligible vanillin conversion, while at 180 °C, the outcome was comparable to that observed at 150 °C, consistently producing only vanillyl formamide (**2a**).

Overall, the best conditions found in this study to perform the synthesis of vanillyl formamide via the extrusion-assisted Leuckart process are those summarized in Scheme 3.

**Scheme 3:**
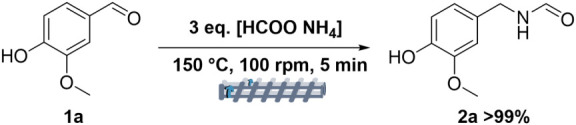
Schematic Representation of the Extrusion-Assisted Leuckart Reaction of Vanillin (**1a**) and Ammonium Formate, under Optimized Conditions (Q: 3; T: 150 °C; t: 5 min; 100 rpm)

The reaction was a thermal-mechanochemical-assisted process, where both the temperature and the mechanical action/energy contributed synergistically to achieving an extremely rapid, quantitative, and selective reaction.

Moreover, compared to conventional solvent-based Leuckart protocols that typically reported the use of a large excess of ammonium formate, from 5 to 100 equiv with respect to the carbonyl reactant,^26^ the reactive extrusion allowed a drastic reduction of the ammonium formate:vanillin molar ratio (*Q* = 3), which was crucial toward a more efficient use of resources/reactants and waste minimization.

Two additional experiments were carried out with the aim of comparing the reactive extrusion to a batch procedure. The tests (batch A and batch B) were run simultaneously in two glass flasks, both loaded with a mixture of vanillin and ammonium formate in a 1:3 molar ratio and heated at 150 °C for 5 min (conditions of Scheme 3), under magnetic stirring. For batch A, neat conditions were used: although the temperature of 150 °C was above the melting point of both vanillin and ammonium formate, the viscosity and consistency of the molten mixture were such that stirring was not possible, thereby hindering the reaction itself. No conversion was achieved not only after 5 min but even after 1 h. For batch B, DMSO (15 mL) was used as the solvent. Full conversion of vanillin was observed toward a mixture of primary, secondary, and tertiary amines including vanillyl amine (12%), compounds **5a** (54%) and **6a** (34%), respectively (Scheme 4; see also Figure S7 for structures assignment).

**Scheme 4:**
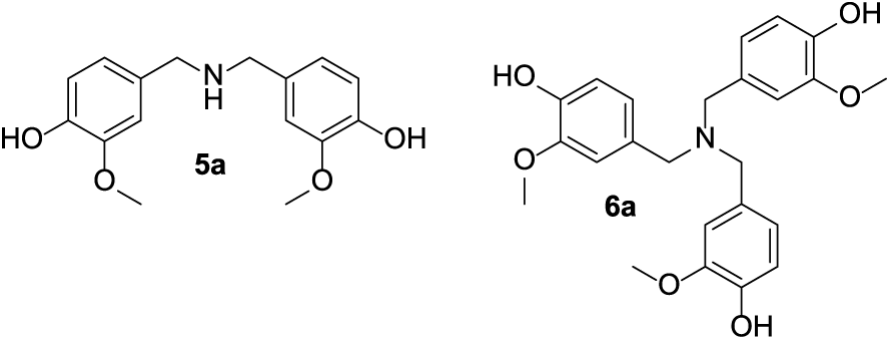
Structures of the Secondary and Tertiary Amines, **5a** and **6a**, Formed in the Batch Reductive Amination of Vanillin with Ammonium Formate (1:3 Molar Ratio) in DMSO (15 mL), Heated at 150 °C for 5 min

These results proved that batch conditions were neither convenient nor practicable for synthesis: not only did the reaction mandatorily require a high-boiling solvent such as DMSO, but even most importantly, it was not selective at all. By contrast, the reactive extrusion was a solventless procedure that enabled the exclusive formation of the amide **2a**. This further supported the advantages of mechanochemical activation.

#### Scale-Up and Green Metrics Evaluation

Conditions of Scheme 3 were considered for a preliminary investigation of the scale-up feasibility of the protocol. An experiment was carried out by increasing 5-fold the amount of vanillin, from 5 mmol (0.76 g) to 25 mmol (3.8 g) and keeping the ammonium formate:aldehyde molar ratio set to 3. The test was successful: amide **2a** was achieved with a 99% selectivity at complete conversion. The corresponding space-time yield (STY), defined as the mass of product obtained per unit reactor volume and per unit time, was 2.74 kg·L^–1^·h^–1^, based on a reactor volume of 0.02 L and a reaction time of 5 min. Albeit this aspect was not further inspected, the result proved that the extrusion-based protocol was viable for a multigram synthesis and could be possibly designed for applications beyond the laboratory practice.

CHEM21 toolkit’s Zero Pass assessment was used to evaluate the green metrics, particularly based on selectivity (S), conversion (C), optimum efficiency (OE), yield (ε), atom economy (AE), and reaction mass efficiency (RME) of the extrusion-assisted reaction of vanillin with ammonium formate.^27^ The results are listed in Figure 4.

**Figure 4:**
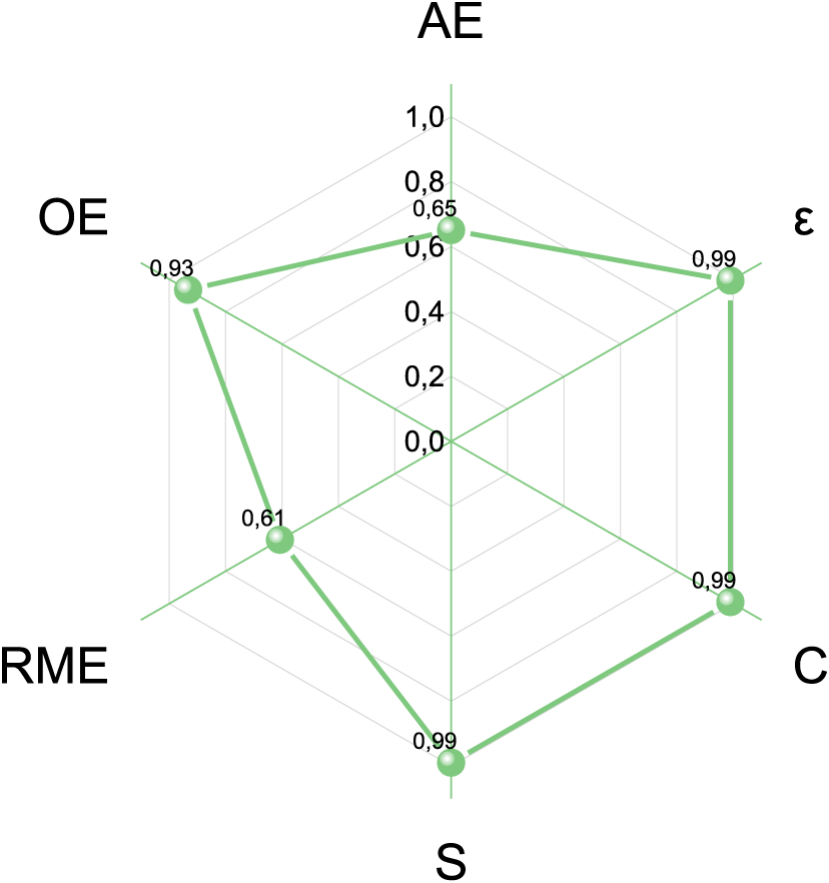
Hexagon radial chart for the evaluation of the metrics of the synthesis of vanillin formamide via the extrusion-assisted reaction of vanillin and ammonium formate. Conditions of Scheme 3 (Q: 3; T: 150 °C; t: 5 min; 100 rpm).

The high values for S, C, OE, and ε (0.99, 0.99, 0.93, and 0.99, respectively) reflected the good synthetic performance of the protocol, while the relatively low AE and RME values were due to the stoichiometry of the involved reactions, specifically the formation of side products as carbon dioxide and water originated during the overall process (Scheme 1). Nevertheless, compared to traditional liquid phase-based methods, the reactive extrusion still appeared far superior in terms of lower consumption of reagents and production of waste, not only for the minimization of the ammonium formate:vanillin molar ratio (*Q* = 3), but also for the absence of solvents.

#### Substrate Scope

The general applicability of the reactive extrusion for amide preparation was investigated by exploring the reaction of ammonium formate with four different aldehydes. These were chosen among solid compounds such as *ortho*-vanillin (**1b**), 5-methylsalicylaldehyde (**1c**), 4-chlorobenzaldehyde (**1d**), and 4-bromobenzaldehyde (**1e**). Control tests were initially carried out under the conditions used for vanillin (Scheme 3). However, the conversion of aldehydes was not satisfactory, not exceeding 55%. Further experiments proved that the reaction was more conveniently controlled by operating at a lower screw rotation speed of 50 rpm and a reaction time prolonged to 15 min. The amount of the carbonyl reactant and the ammonium formate:aldehyde molar ratio were kept unchanged to 5 mmol and *Q* = 3, respectively, as in Scheme 3. The results are reported in Table 1. For convenience, the table also includes the reaction of vanillin (entry 1) run under the same set of conditions as used for the other aldehydes.

**Table 1:**
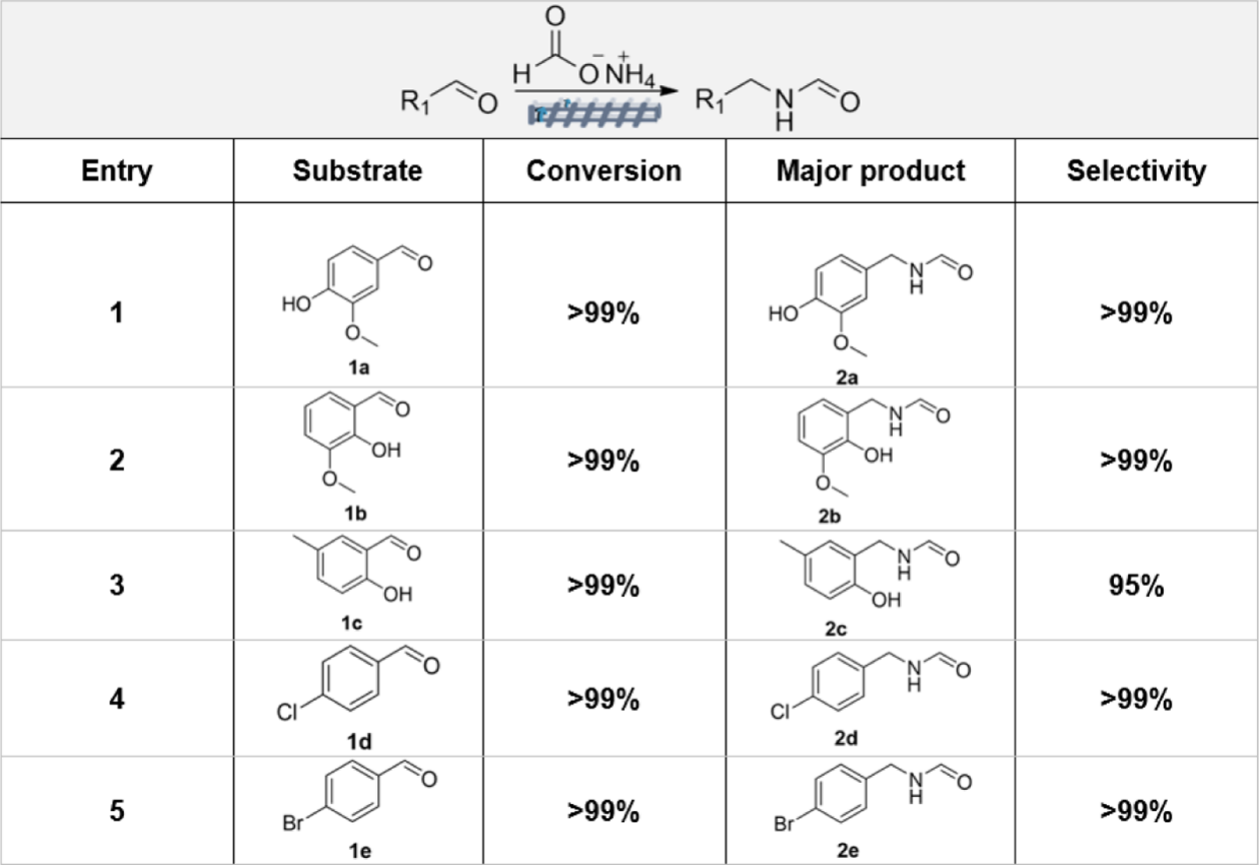
Substrate Scope of the Amide Synthesis via an Extrusion-Based Leuckart-Type Approach^a^

a Experiments were run by reactive extrusion. Conditions: *T* = 150 °C; *t* = 15 min, screw rotation speed = 50 rpm; reactant aldehyde = 5 mmol; ammonium formate:aldehyde molar ratio = 3.

*Ortho*-vanillin, 4-chlorobenzaldehyde and 4-bromobenzaldehyde yielded the corresponding amides **2b**, **2d**, and **2e** with excellent conversion and selectivity (>99%), equally high as those achieved for vanillin (entries 2, 4, and 5). The reasons why reaction conditions (rotation speed and reaction time) had to be changed compared to Scheme 3 still require further investigations, but a hypothesis was formulated based on two aspects: (i) the remarkably lower melting points of the substrates (**1b**: 41 °C; **1d**: 47 °C; **1e**: 57 °C) compared to vanillin (83 °C).^28^ This could affect the lubrication of the reaction mixture and the contacts of the reagents within the screw barrel. A similar effect was described in a previous study on the Knoevenagel condensation of vanillin and some of its derivatives (veratraldehyde and 5-bromovanillin) with barbituric acid, carried out via twin-screw extrusion^29^ (ii) the decrease of the screw speed (from 100 to 50 rpm). If this resulted in a lower level of mixing of the material and a lower supply of shear and mechanical energy to the reaction mixture, it also increased the residence time that helped to complete the reaction. Interestingly, in a study on the continuous extrusion for the synthesis of organic dyes, it was noticed that product yields improved by a slow screw-rotational speed and a reduced feed rate.^30^

The reaction of 5-methylsalicylaldehyde (**1c**, mp = 56 °C)^28^ was also quantitative, but a slightly poorer selectivity toward amide **2c** was noticed, not exceeding 95% (entry 3). In this case, *N*,*N*-bis­(2-hydroxy-5-methylbenzyl)­formamide (**3c**: 5%) was detected as a secondary product resulting from a condensation reaction analog to that described for the case of vanillin in Scheme 2 (further details are reported in Scheme S1). Two additional tests were performed using ketone substrates (benzophenone and 2,4-dihydroxybenzophenone) but the results showed no conversion, probably due to the lower electrophilicity of the carbonyl carbon in ketones in comparison with aldehydes and the steric hindrance given by the phenyl groups of benzophenone.

Overall, the results demonstrated that the extrusion-assisted protocol was effective for a variety of substrates, but adjustments in the reaction parameters, particularly, the screw speed and reaction time, were necessary: the most reliable conditions for a broader substrate applicability were identified at 50 rpm and 15 min. These conditions also proved suitable for vanillin in accordance with Figure 3A.

### Amine Synthesis via an Extrusion-Based Leuckart Reaction

The Leuckart reaction via continuous extrusion was further investigated with the aim of broadening the scope of the protocol beyond the synthesis of amides. The preparation of amines was considered according to the strategy summarized in Scheme 5.

**Scheme 5:**
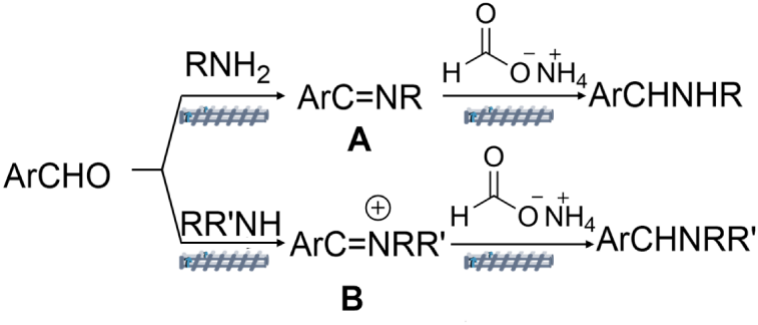
Schematic Illustration of the Reactive Extrusion Strategy for the Synthesis of Amines

The goal was the initial conversion of a carbonyl compound, specifically an aldehyde, into an imine or an iminium ion, A or B, by the reaction with a primary or a secondary amine (Scheme 5, top and bottom). Thereafter, in the extrusion barrel, these intermediate species were made to react with ammonium formate as a reductant to produce a secondary or a tertiary amine, respectively.

Initial experiments were carried out by loading the extruder with an equimolar mixture of vanillin and morpholine as a model secondary amine (5 mmol each) and ammonium formate (3 mol equiv, 15 mmol). At 100 °C and 50 rpm, tests were unsuccessful: even after 60 min, the desired amine, 2-methoxy-4-(morpholino methyl)­phenol, was detected in a negligible amount (**4a**: < 5%), while the reaction mostly halted to the formation of the iminium ion intermediate, 4-(4-hydroxy-3-methoxybenzylidene)­morpholin-4-ium (**4a′**) (further details are in Figure S21).

Since the reducing activity of ammonium formate was apparently not enough for the second step of Scheme 5, we were prompted to design further experiments by introducing a commercial heterogeneous catalyst for hydrogenation reactions, specifically Pd/C (100 mg), which was expected to enhance the hydride donation capability of formic acid.^31^ The results are listed in Table 2. For comparison, the table also includes the reaction outcome without any metal catalyst (entry 1).

Under the same conditions as for entry 1, the presence of Pd/C did induce a remarkable improvement of the process and proved the feasibility of the reactive extrusion protocol: the desired tertiary amine **4a** was achieved with 83% selectivity at a 97% conversion (entry 2). Other coproducts were vanillin amine (<1%) derived from the reaction of vanillin and ammonia originated by the dissociation of ammonium formate, an imine species (ca. 16%) derived from the condensation of vanillin and vanillin amine, and the corresponding amine 2,2′-dimethoxy-4,4′-(2-aza-propanediyl)-diphenol (<1%); further details on these compounds are in the Supporting Information. Sodium formate was investigated as an alternative reducing agent other than ammonium formate in a test analogous to entry 2 of Table 2, however, we did not observe the formation of product **4a**. We believe that the reason is directly related to the mechanism of the reaction: while sodium formate is stable up to 250 °C, and not active itself as a reductant, ammonium formate decomposes into formic acid and ammonia at *T* > 100 °C, and formic acid can then undergo dehydrogenation to generate an active hydride donor.^19^

Additional tests demonstrated that by decreasing the temperature, vanillin provided the iminium ion **4a′**, but no reduction occurred: at 50 °C, the conversion was 42% without any trace of **4a** (entry 3; other effects of the reaction temperature are reported in the Supporting Information). Moreover, at 100 °C, halving the reaction time from 60 to 30 min, resulted in almost no changes in the conversion (92%), while the **4a** selectivity did not exceed 76% (entry 4). This suggested that vanillin was substantially consumed in the first 30 min, but the process had to be prolonged up to 1 h, to complete the transformation/reduction of intermediate species. Finally, an influence of the ammonium formate:vanillin molar ratio (*Q*) was observed. At 100 °C, after 1 h, the use of an equimolar amount of reagents (*Q* = 1) brought about a decrease of the selectivity to 74%, albeit with a good conversion (94%) (entry 5). Doubling the *Q* ratio resulted in an almost quantitative reaction and an increased selectivity of 81% (entry 6). These findings were consistent with the need of a threshold amount of ammonium formate corresponding to a *Q* ratio in the range of 2–3, to allow a satisfactory formation of the amine **4a**.

#### Scale-Up and Green Metrics Evaluation

A further analysis based on the CHEM21 approach illustrated the trade-off between reducing reagent excess and optimizing reaction efficiency (details are in Figure S35): increasing *Q* had minimal effects on conversion, selectivity, and optimum efficiency, but it impacted on decreasing RME due to the generation of byproducts (ammonia, carbon dioxide, and water), and in general, to the lower efficiency on the resource use. Furthermore, Table S1 presents the E-factor (E) and Process Mass Intensity (PMI) for the studied processes. The Leuckart-type reductive amination with *Q* = 1 achieves the lowest E-factor (0.50) and PMI (1.50). Notably, all values remain well below typical solution-phase syntheses, where PMI often exceeds 10 and E-factors can reach double digits, underscoring the potential of these mechanochemical approaches for greener synthesis.

Overall, as noticed for the preparation of amides, the interplay between the reaction temperature, the mechanical energy supplied during extrusion, and the reactant ratio was also crucial for the mechanochemical-assisted synthesis of amines.

The reactive extrusion also proved to be excellent for a multigram synthesis. Under the conditions of entry 2 in Table 2, an experiment carried out by increasing 5-fold the amount of vanillin, from 5 mmol (0.76 g) to 25 mmol (3.8 g) (ammonium formate:aldehyde molar ratio, *Q* = 3; Pd/C = 350 mg), yielded the amine **4a** with a selectivity (90%) even greater than that achieved on a smaller scale (83%, Table 2), at complete conversion. Moreover, the space-time yield (STY) was 0.252 kg·L^–1^·h^–1^, based on a reactor volume of 0.02 L and a reaction time of 1 h.

This part of the study was concluded by comparing reactive extrusion to a batch procedure. The (batch) test was carried out at the same *T*, reactants’ molar ratio, and time reported in entry 5 of Table 2. A mixture of vanillin (5 mmol), morpholine, and ammonium formate in a 1:1:3 molar ratio, respectively, and Pd/C (100 mg) was loaded in a glass flask, heated to 100 °C under magnetic stirring, and set to react for 60 min. Under such conditions, the reaction mixture melted. The conversion of vanillin was high (90%), but the batch reaction failed in providing the amine **4a**. The intermediate iminium ion **4a′** was the only detected species. This result further highlighted the potential of the reactive extrusion to design synthetic strategies that offered both access to products (amines) otherwise not obtained by conventional batch protocols and the typical benefits of continuous-flow methods. Moreover, recycling experiments were designed to assess the stability and reusability of the metal catalyst. Once a reaction was completed under the conditions of entry 2 in Table 2, Pd/C was recovered by filtration, washed with acetone, dried overnight, and reused for another reaction. The overall sequence was repeated once more for a total of three subsequent uses (the starting test + two recycles) of the same catalyst batch. The analysis of the reaction mixtures collected at the outlet of the extruder proved that the catalyst fully preserved its performance after two recycles: the conversion of vanillin was quantitative and selectivity toward product **4a** was 85%.

#### Substrate Scope

The scope of the reactive extrusion implemented for the synthesis of amine **4a** was further investigated by exploring different substrates, including both aldehydes and amines. Particularly: (i) the reaction of morpholine and ammonium formate was carried out with two solid aldehydes as *ortho*-vanillin (**1b**) and 5-methylsalicylaldehyde (**1c**), and (ii) the reaction of vanillin with ammonium formate was extended to two secondary amines as diethanolamine (**1f**) and *N*-methyl-*p*-anisidine (**1g**) and three primary amines, both aromatic and aliphatic species, as anisidine, *p*-aminophenol, and decylamine.

Experimental conditions were those of entry 2 in Table 2 (100 °C; equimolar mixture of aldehyde and amine, 5 mmol of each reagent; ammonium formate: aldehyde molar ratio of 3; Pd/C, 100 mg; 60 min, 50 rpm). Figure 5 schematizes the apparatus and the operating procedures used for the reaction.

**Table 2:**
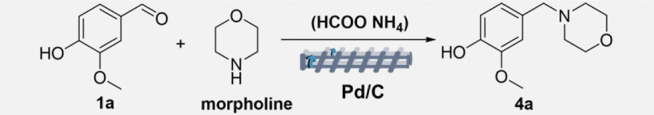
Parametric Analysis of the Reaction of Vanillin, Morpholine, and Ammonium Formate under Reactive-Extrusion^a^

Entry	Cat.	*T* (°C)/*t* (min)	*Q* (mol:mol)	Conv. (1a, %)	Sel. 4a (%)
1	-	100/60	3	91	-
2	Pd/C	100/60	3	97	83
3		50/60	3	42	-
4		100/60	3	92	76
5		100/60	1	94	75
6		100/60	2	99	81

a *Q* = vanillin:ammonium formate molar ratio. Other conditions: vanillin (5 mmol), morpholine (5 mmol), rotation speed: 50 rpm; Pd/C (100 mg).

**Figure 5:**
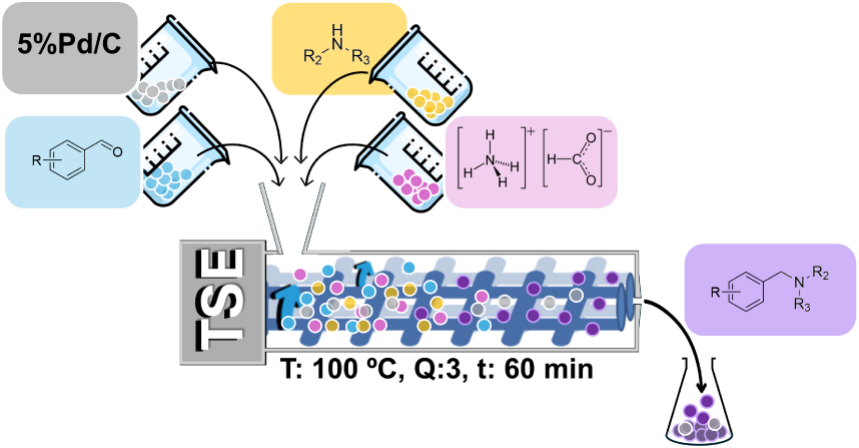
Schematic representation of the mechanochemically assisted synthesis of amines via a catalytic Leuckart reaction. Reactions were carried out using vanillin, morpholine, and ammonium formate as model substrates.

Results are reported in Table 3. For convenience/comparison, the table also includes the reactive extrusion of vanillin and morpholine (entry 1).

**Table 3:**
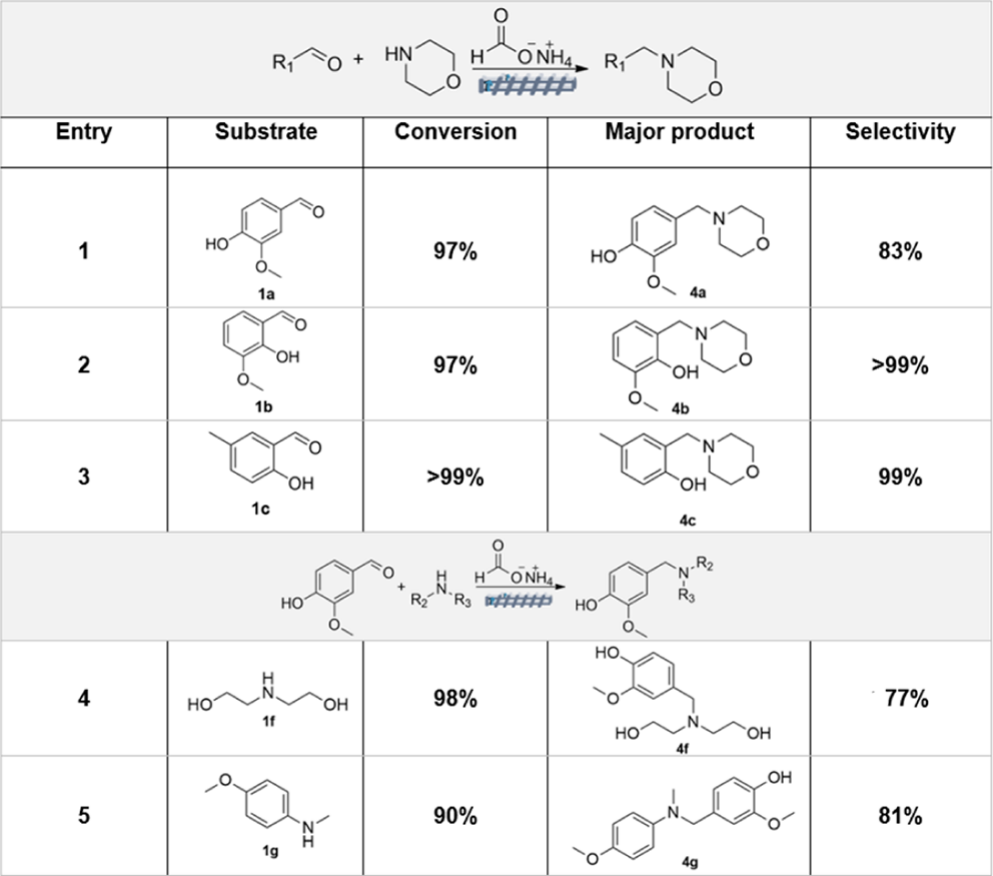
Substrate Scope of the Synthesis of Amines via Reactive Extrusion Based on the Leuckart Reaction^a^

a Experiments were run under the conditions set for reactive extrusion: *T* = 100 °C; *t* = 60 min, screw rotation speed = 50 rpm; ammonium formate:aldehyde molar ratio = 3.

The reaction of *ortho*-vanillin (**1b**) and 5-methylsalicylaldehyde (**1c**) with morpholine proceeded even better than that of vanillin: in both cases, an excellent conversion and amine selectivity of 97% and 99%, respectively, were reached (entries 2 and 3). This outcome, particularly the high selectivity, was ascribed to an *ortho* effect by which the hydroxy substituent stabilized the adjacent carbonyl through hydrogen bonding, thereby mitigating the reactivity and improving the selective conversion to the desired amine.

The use of other amines provided largely different results. Irrespective of their aromatic or aliphatic nature, primary amines proved ineffective to achieve the sequence of Scheme 5 (top): the corresponding imines (step 1) were obtained in all cases, but the imine-to-amine reduction (step 2) did not occur, not even in trace amounts. By contrast, as noticed for morpholine, the tested secondary amines successfully allowed the formation of the expected tertiary amines. The reactions of vanillin with both diethanolamine (**1f**) and *N*-methyl-*p*-anisidine (**1g**) proceeded comparably to those with morpholine, thereby confirming the robustness of the reactive extrusion: high conversions of 98% and 90% were obtained along with amine selectivity of 77% and 81%, respectively (entries 4–5).

The different behaviors of primary and secondary amines were unclear. Though, a hypothesis was formulated based on the nature of the intermediate species:^32,33^ compared to imines that, once obtained from the primary amines in the extrusion barrel, did not react anymore, it was assumed that the inherent higher reactivity of iminium ions (generated from secondary amines) lowered the energy barrier for their subsequent Pd/C-catalyzed reduction, thereby making their conversion to the observed tertiary amines. Whatever the reasons, the failure to convert primary amines to the desired products identified a limitation of the designed protocol. This aspect will require further investigation, which is, however, beyond the scope of this work.

## Conclusions

This study has successfully developed a new mechanochemical method for the synthesis of amides and amines via the Leuckart reaction carried out by extrusion. A parametric analysis of the reaction of ammonium formate and vanillin chosen as a model case for the amide synthesis has revealed that the combined effect of thermal and mechanical energy allowed an exceptionally fast and scalable transformation. In only 5 min at 150 °C, the aldehyde was quantitatively converted into the desired amide through a continuous process feasible on a multigram scale. The protocol was equally efficient for a small but representative library of solid aromatic aldehydes (4 examples) that proved the concept, yielding the corresponding benzyl-type amides as exclusive products.

The reactive extrusion proved versatile also for the synthesis of tertiary amine via a multistep sequence, where the initial condensation of an aldehyde and a secondary amine originated an iminium ion as an intermediate, which in turn was reduced by ammonium formate to produce a tertiary amine. In this case, a metal catalyst as Pd/C was required to activate ammonium formate, particularly to promote a hydride transfer from formic acid to the intermediate species. Conditions for extrusion were therefore different from those for amides, and in general, prolonged reactions of up to 1 h were necessary to prepare amines. Nonetheless, complete conversions and selectivity in the range 80–99% were reached for diverse reagents, including three aldehydes and three amines. A limitation of the protocol was observed by using primary amines, both aromatic and aliphatic ones: in this case, the reaction produced the corresponding imines as intermediates, which did not react further, i.e., they were not reduced to the expected secondary amines.

Whatever the conditions and within the above-described limits, several aspects highlighted how the reactive extrusion was comparatively far more efficient and greener than liquid-phase processes for the Leuckart reaction. Among them, the most relevant were: (i) the absence of solvents; (ii) the use of an ammonium formate:carbonyl reagent molar ratio (*Q*) not exceeding 3; (iii) the extremely fast kinetics (5–60 min); and (iv) the continuous mode ideal to scale-up applications. Conventional transformations in solution, instead, typically required a *Q* of up to 100 and several hours, if not days, for completion.

In conclusion, it must be considered that this work required remarkable effort for the design and the engineering of a new synthetic procedure and could hardly be exhaustive from all points of view. However, there is no reason to believe that the proposed mechanochemical-based methodology cannot be extended to a broader variety of reagents and products (more suited, for example, to applications as drugs or material development), providing that these compounds fulfill the requisites for reactive extrusion as solid and thermally stable compounds. Moreover, despite the growing interest in mechanochemical synthesis, its underlying mechanistic foundations are still not fully understood. In this regard, future studies on the application of computational tools such as DFT calculations could provide deeper details, also related to the translation from small-scale ball milling^34^ to continuous reactive extrusion.^35^ These prospects require additional steps of development and optimization, which are beyond the scope of the present work and will be the object for future investigations.

## Supplementary Material



## Data Availability

All relevant data are within the manuscript and its Supporting Information.

## Notes

Proposed mechanistic schemes (Leuckart reaction, hydrolysis pathways, reductive amination with auxiliary amines); detailed characterization data (^1^H and ^13^C NMR, MS spectra) for formamides 2a–2e, vanillylamine, and morpholine-derived products 4a–4g, as well as iminium intermediates; additional LC-MS and GC-MS analyses of side products and imines; experimental details on product workup; and green metrics evaluation of extrusion-assisted syntheses, including CHEM21 Zero Pass assessment, E-factor and PMI calculations, with a radial chart comparison of reaction conditions (PDF)
