# Supplementary material for: Mechanochemical Extrusion for Sustainable Synthesis of Amides and Amines via the Leuckart Reaction
Source: ACS Sustain Chem Eng. 2025 Oct 7;13(41):17412–21. doi: 10.1021/acssuschemeng.5c06980 (PMC12541804; doi:10.1021/acssuschemeng.5c06980)
Supplement: Supplementary file 1 [file sc5c06980_si_001.pdf]

Supporting information

Mechanochemical Extrusion for Sustainable Synthesis of Amides and Amines via the Leuckart Reaction

Francesco Zorzetto,[a] Alvise Perosa,[a] Daily Rodriguez-Padron[a]* and Maurizio Selva*[a]

[a] [a] Department of Molecular Science and Nanosystems, Ca' Foscari University of Venice, Via Torino 155, 30175 – Venezia Mestre, Italy
E-mail: D.R.P. daily.rodriguez@unive.it M.S. selva@unive.it

Abstract: An extrusion-based, solvent-free method was developed for the synthesis of amides and amines via the Leuckart reaction, representing the first adaptation of this classical transformation into a continuous mechanochemical platform. The production of amides was demonstrated through the model reaction between ammonium formate and vanillin, leading to vanillyl formamide, while the synthesis of tertiary amines was investigated using ammonium formate, vanillin, and morpholine as reactants. A thorough parametric analysis revealed that the mechanochemical-based strategy allowed extremely fast processes: at 100-150 °C, reactions were quantitative in 5-15 min with product selectivity >99% for vanillin formamide and up to 83% for the corresponding tertiary amine. An investigation of the substrate scope (four examples) confirmed the robustness of the protocol for both reaction types. Benzyl-type amides and tertiary amines were synthesized with high conversion and selectivity, exceeding 95%, across different aldehydes. Moreover, morpholine could be successfully replaced by other secondary amines (diethanolamine and N-methyl-p-anisidine), thereby further demonstrating the versatility of the reactive extrusion for the preparation of amines. Overall, this work underscores the novelty and impact of solvent-free continuous extrusion, establishing it as a scalable and sustainable alternative to liquid-phase synthesis, and marking a significant step forward in green chemistry practices.

Contents

Scheme S1.. *N*,*N*-bis(4-hydroxy-3-methoxybenzyl) formamide (3a) detected in some tests run under the conditions of Figure 2 2

Scheme S2.. Proposed mechanism for the synthesis (2-hydroxy-3-methoxybenzyl)formamide (vanillyl formamide, 2a) via the Leuckart reaction……………………………………………………………………………2

Scheme S3.. Acid hydrolysis of the amide 2a to vanillylamine and formic acid…………………………….…2

Scheme S4.. Proposed mechanism for the synthesis (2-methoxy-4-(morpholinomethyl)phenol, 4a) via the Leuckart-type reductive amination reaction with an auxiliary amine (morpholine)……………………..3

Products characterization..3

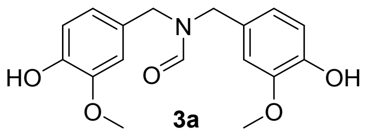

Scheme S1. *N*,*N*-bis(4-hydroxy-3-methoxybenzyl) formamide (**3a**) detected in some tests run under the conditions of Figure 2.

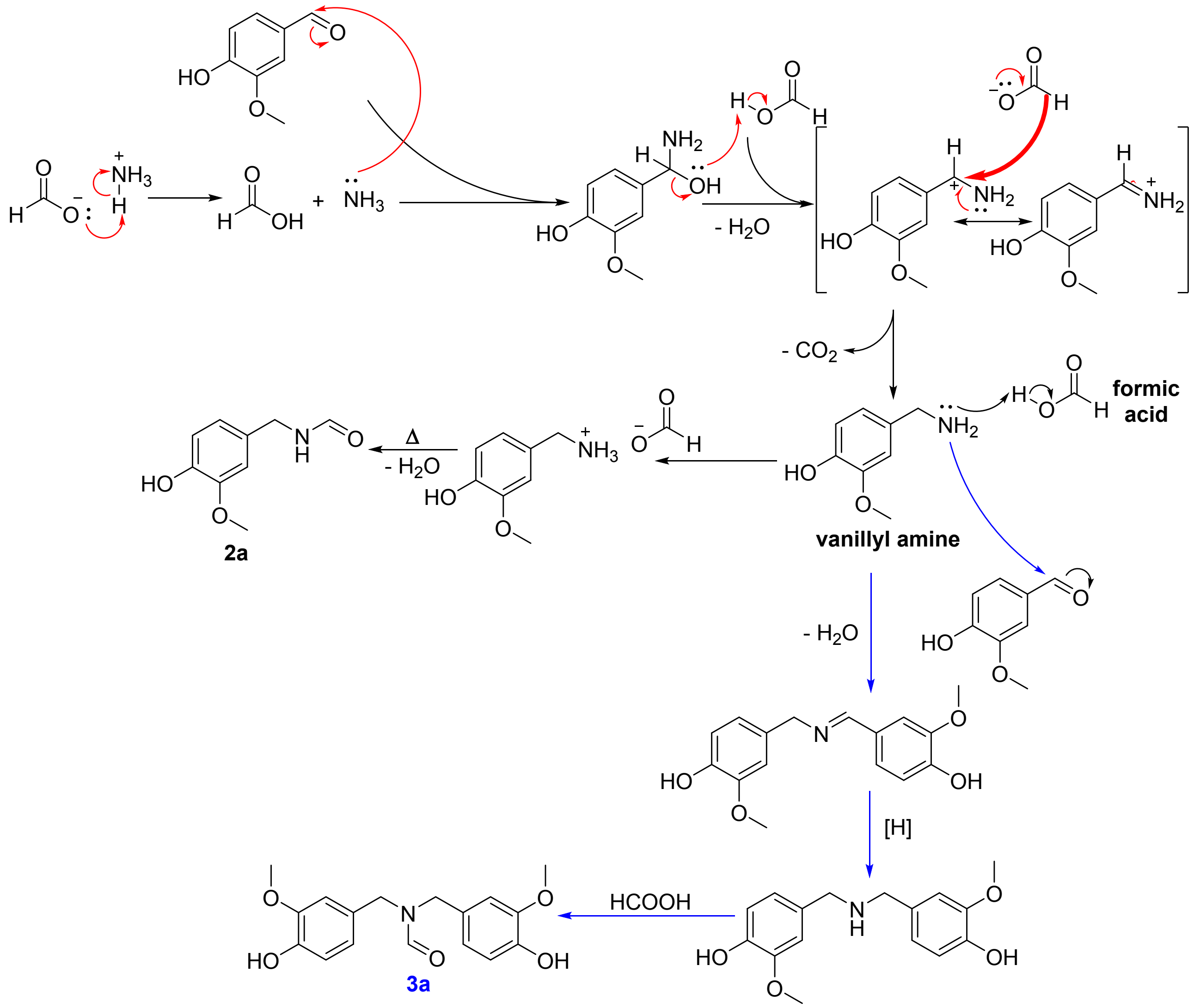

Scheme S2. Proposed mechanism for the synthesis (2-hydroxy-3-methoxybenzyl)formamide (vanillyl formamide, **2a**) via the Leuckart reaction.

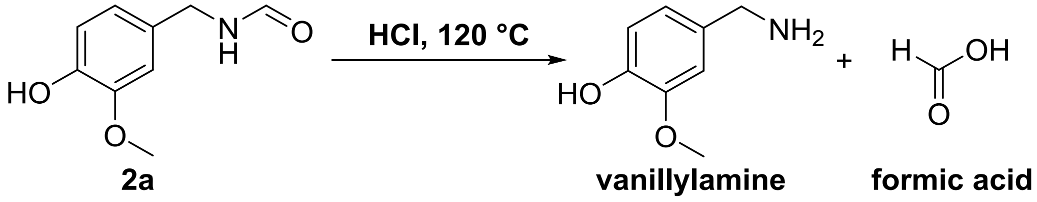

Scheme S3. Acid hydrolysis of the amide 2a to vanillylamine and formic acid.

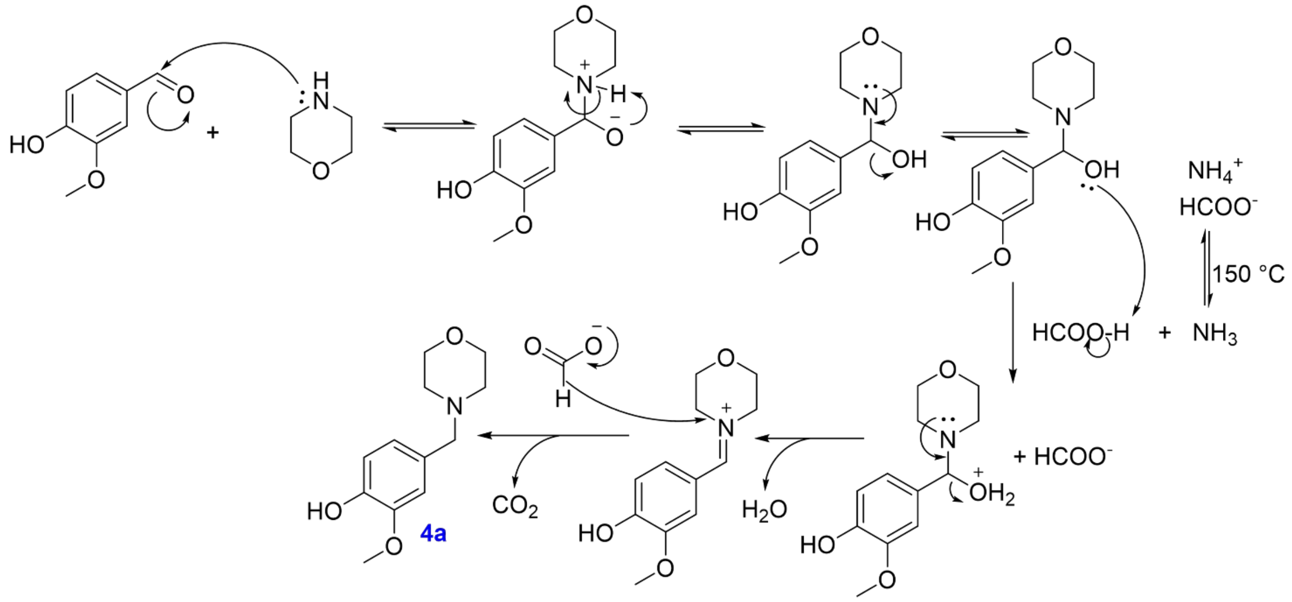

Scheme S4. Proposed mechanism for the synthesis (2-methoxy-4-(morpholinomethyl)phenol, 4**a**) via the Leuckart-type reductive amination reaction with an auxiliary amine (morpholine).

Products characterization

Work-up. Before products characterization, the following work-up procedure was used.

Amides. Once the reaction mixture was extruded, at the outlet of the extrusion barrel, a sample of the solid mixture of ca 100 mg was recovered and allowed to cool to rt.
For MS analyses, 5-10 mg of the solid was dissolved in MeOH (0.5 mL) and injected as such. GC-MS or LC-MS analyses were carried out as described in the experimental section.
For NMR analyses, ca 50 mg of the solid mixture was added with chloroform (10 mL) to precipitate the excess ammonium formate. The suspension was then filtered, and the solution was rotary evaporated to remove the solvent. The dried solid was analysed by NMR without any further purification. The analyses were carried out as described in the experimental section, using CD_3OD as the solvent.

Amines. Once the reaction mixture was extruded, at the outlet of the extrusion barrel, a sample of the solid mixture of ca 100 mg was recovered and allowed to cool to rt.
For MS analyses, 5-10 mg of the solid was suspended in MeOH (0.5 mL) and centrifuged to separate traces of the undissolved catalyst. The homogeneous solution was injected as such. GC-MS or LC-MS analyses were carried out as described in the experimental section.
For NMR analyses, ca 50 mg of the solid mixture was added with chloroform (10 mL) to precipitate the excess ammonium formate which settled along with traces of the heterogenous catalyst. The suspension was then filtered, and the solution was rotary evaporated to remove the solvent. The dried solid was analysed by NMR. The analyses were carried out as described in the experimental section, using $CDCl_3$ as the solvent.
Before NMR characterization, compound **4d** was achieved with selectivity of 77%, thus required further purification. The solid mixture obtained at the outlet of the extrusion barrel was subjected to flash chromatography on silica gel (eluant ethyl acetate: cyclohexane (70:30). After this step, GC-analysis proved that the purity of the product was higher than 95%. **4d** was then analysed by NMR.

All reagents used in this work were ACS grade. Some of them, however, displayed purities below 99%. These included vanillin (97%), ammonium formate (97%), 2-hydroxy-5-methylbenzaldehyde (98%), chlorobenzaldehyde (98%), and N-methyl-p-anisidine (95%). Attempts to further purify these compounds proved unsuccessful. Some unexpected signals observed in some NMR spectra were plausibly due to impurities present in the original reagents.

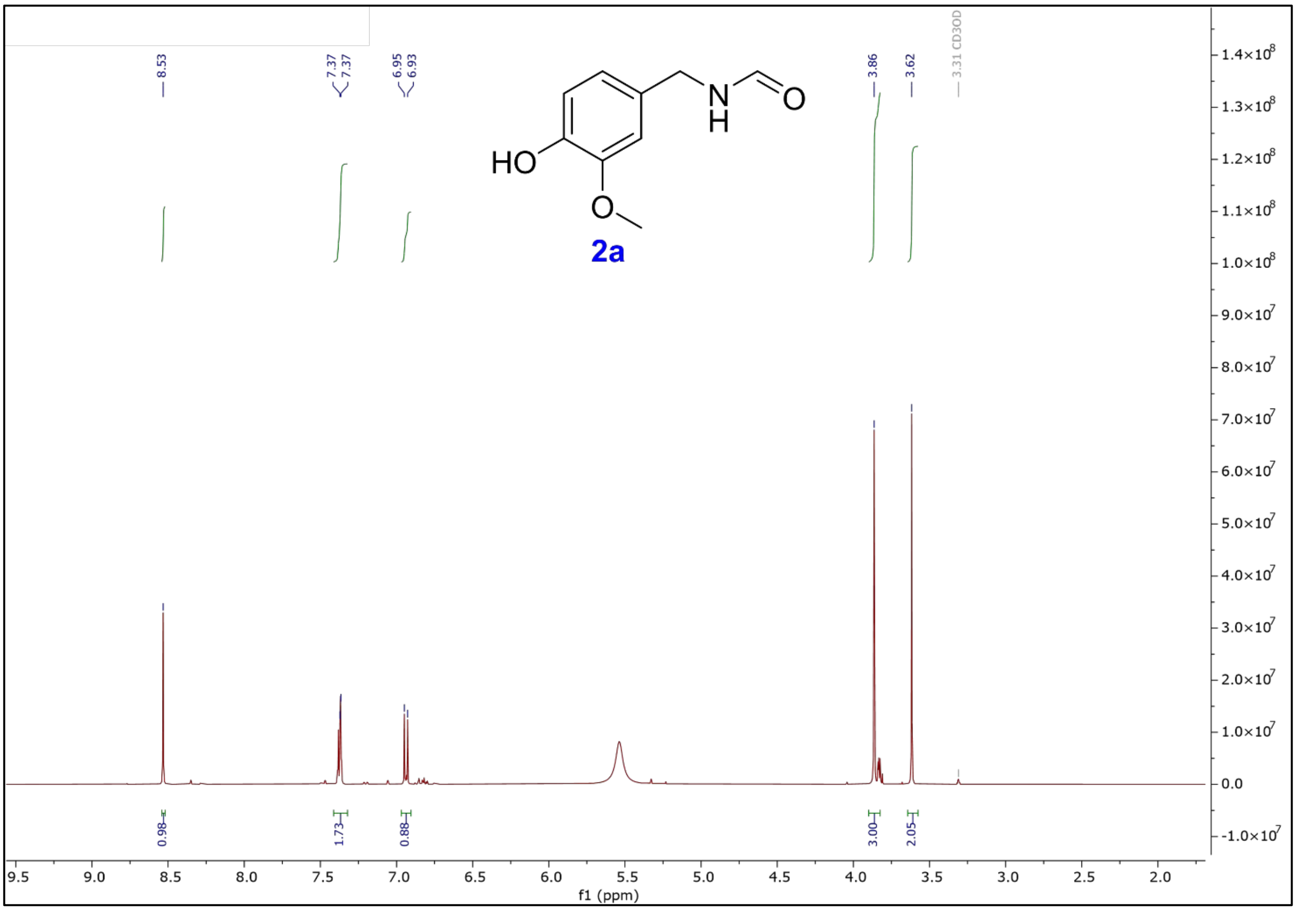

Figure S1: ^{1}H-NMR spectrum of **2a** (vanillylformamide) (400 MHz, 298 K, CD_3OD-d4), δ, ppm: 3.62 s (2H, NCH_2) 3.86 s (3H, OCH_3), 6.93-6.95 m (3H, H_{arom}), 8.53 s (1H, OCH).

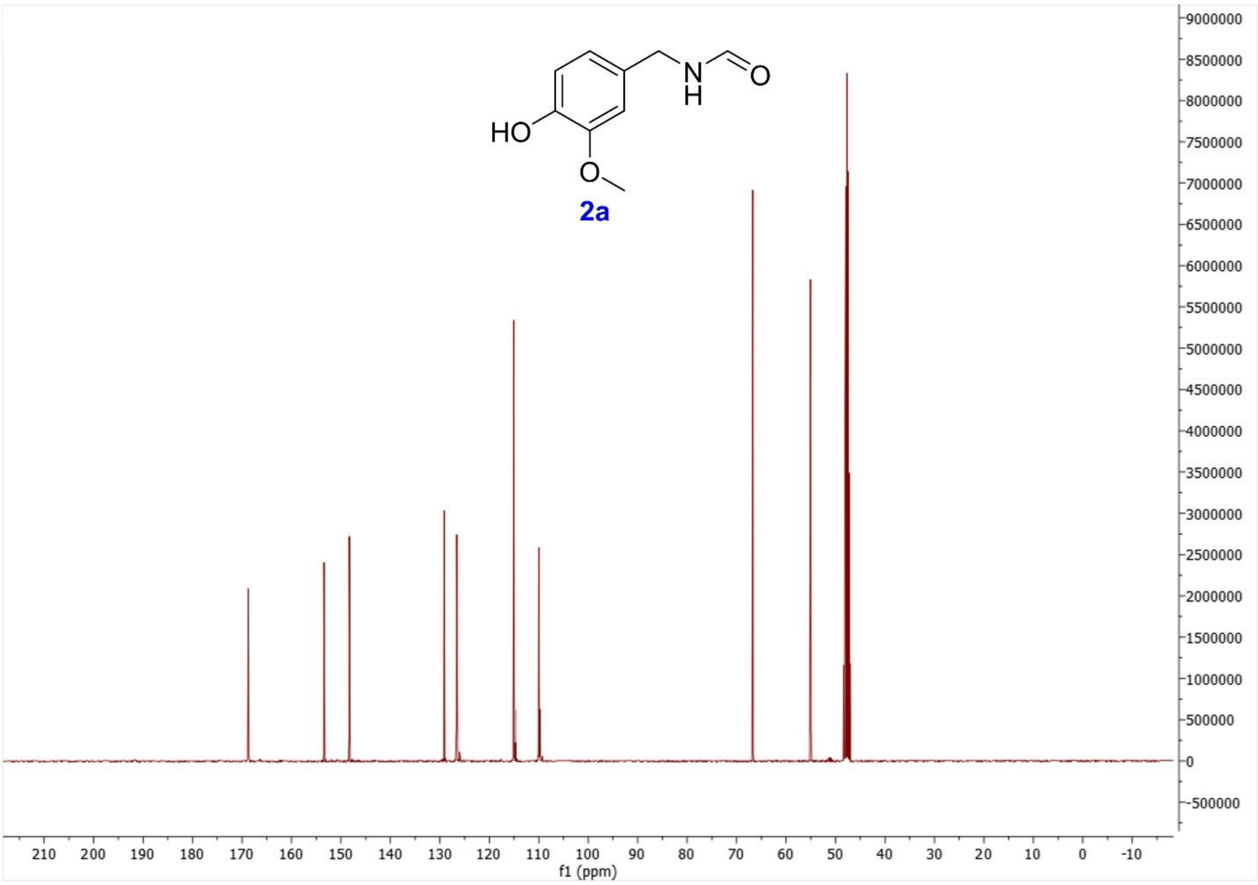

Figure S2: ^{13}C-NMR spectrum of **2a** (vanillylformamide) (400 MHz, 298 K, CD_3OD-d4), δ, ppm 169.7 (C, -NHCHO), 154.0 (C_{arom}), 149.5 (C_{arom}), 129.9 (C_{arom}), 127.0 (C_{arom}), 115.0 (C_{arom}), 110.1 (C_{arom}), 67.0 (C, NCH_2), 55.4.ppm (C, OCH_3).
The NMR characterization of product **2a** has also been previously reported in the literature.[1]

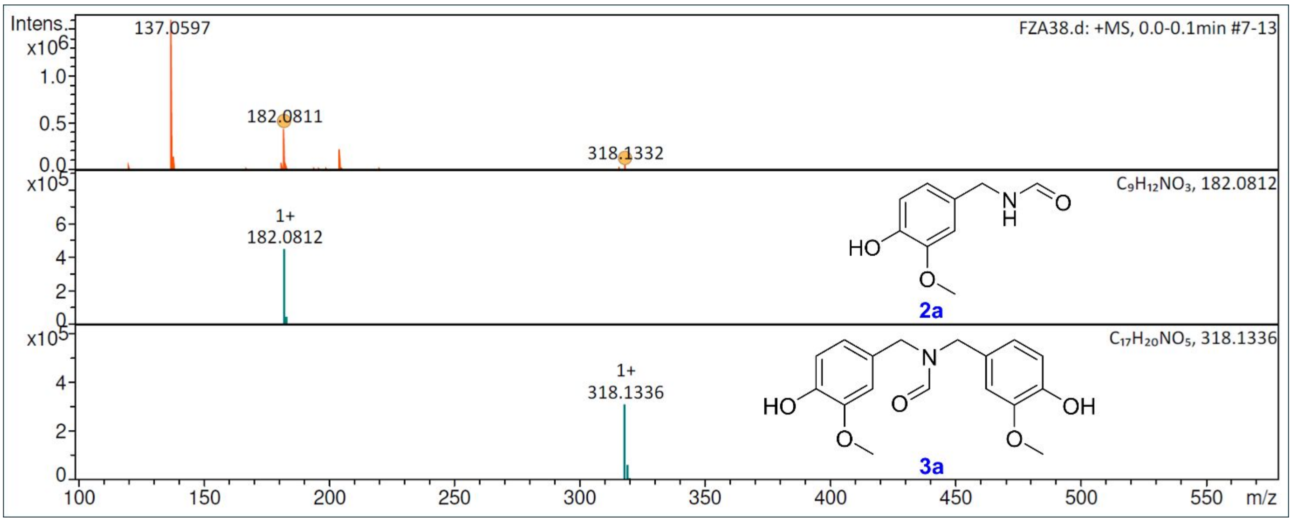

Figure S3: MS-spectra of **2a** (vanillylformamide, m/z=182) and **3a** (m/z=318) obtained in LC-MS.

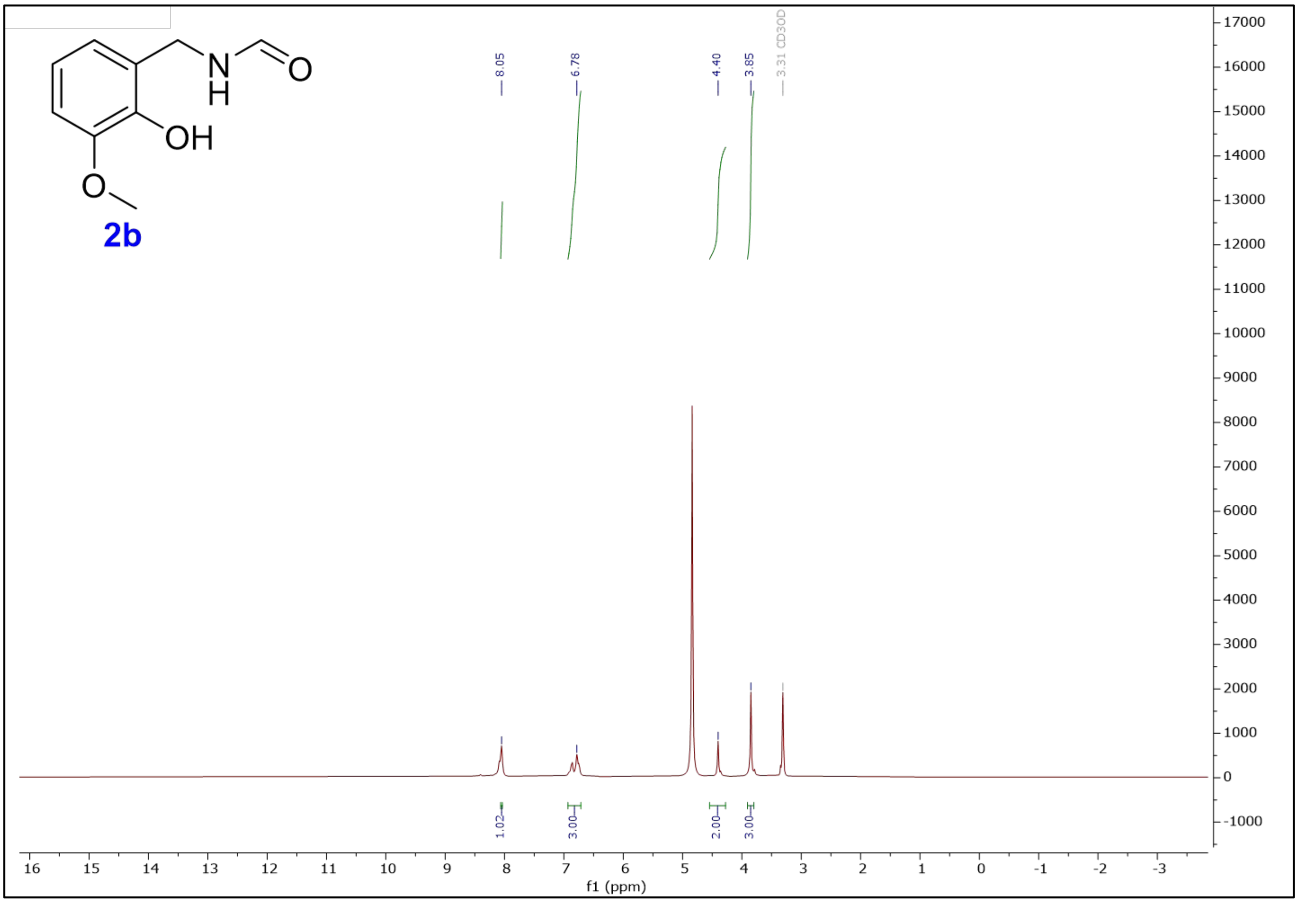

Figure S4: ^{1}H-NMR spectrum of **2b** (o-vanillylformamide) (400 MHz, 298 K, CD_3OD-d4), δ, ppm: 3.85 s (3H, OCH_3), 4.40 s (2H, NCH_2), 6.78 m (3H, H_{arom}), 8.05 s (1H, OCH).

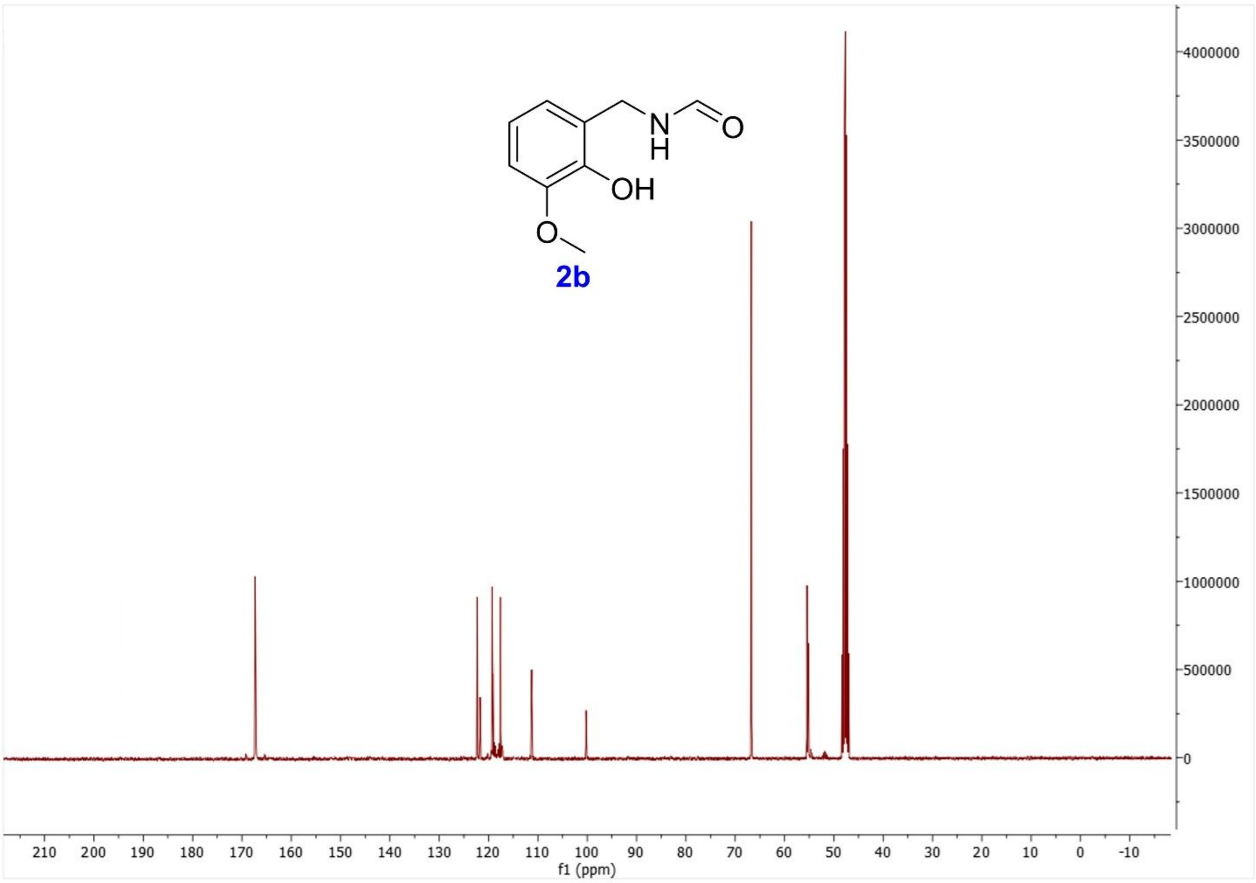

Figure S5: ^{13}C-NMR spectrum of **2b** (o-vanillylformamide) (400 MHz, 298 K, CD_3OD-d4), δ, ppm 168.5 (C, -NHCHO), 123.2 (C_{arom}), 123.0 (C_{arom}), 119.9 (C_{arom}), 118.0 (C_{arom}), 111.4 (C_{arom}), 101.1 (C_{arom}), 66.7 (C, NCH_2), 55.2 ppm (C, OCH_3).
The NMR characterization of product **2b** has also been previously reported in the literature.[2]

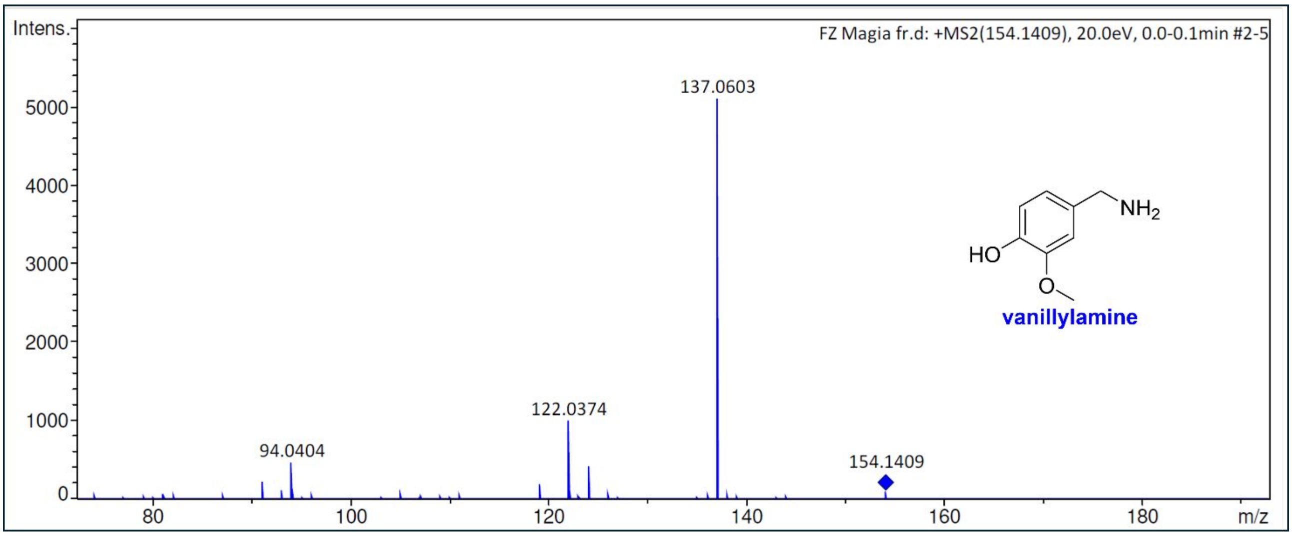

Figure S6: MS-spectrum of vanillylamine (m/z=154) obtained in LC-MS.

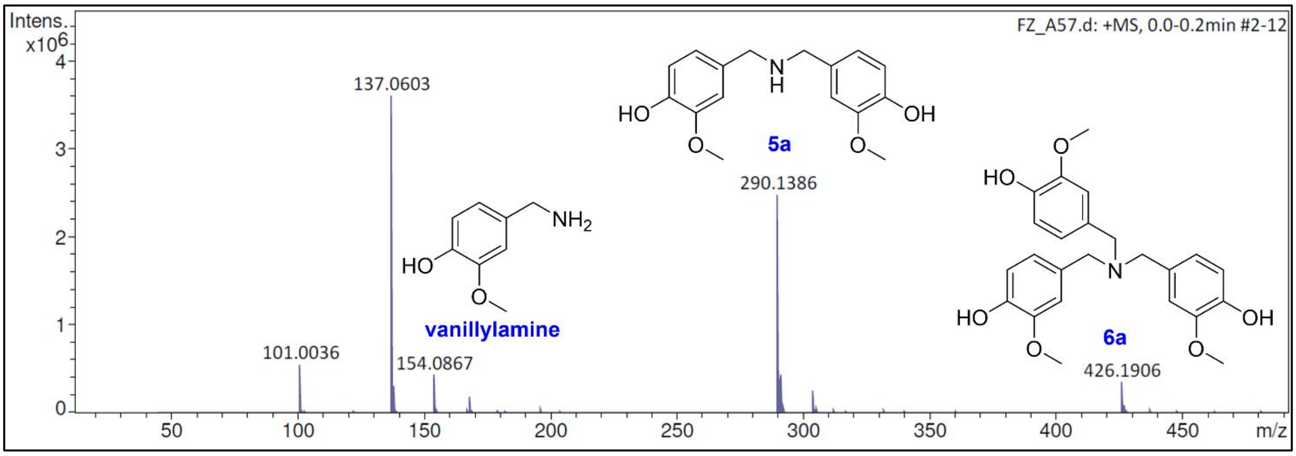

Figure S7: MS-spectra (LC-MS) of products obtained during the batch reaction of vanillin and ammonium formate (molar ratio 1:3) at 150 °C, 5 min of reaction time, in the presence of DMSO as a solvent. **Vanillyl amine** (m/z=154), **5a** (m/z=290) and **6a** (m/z=426).

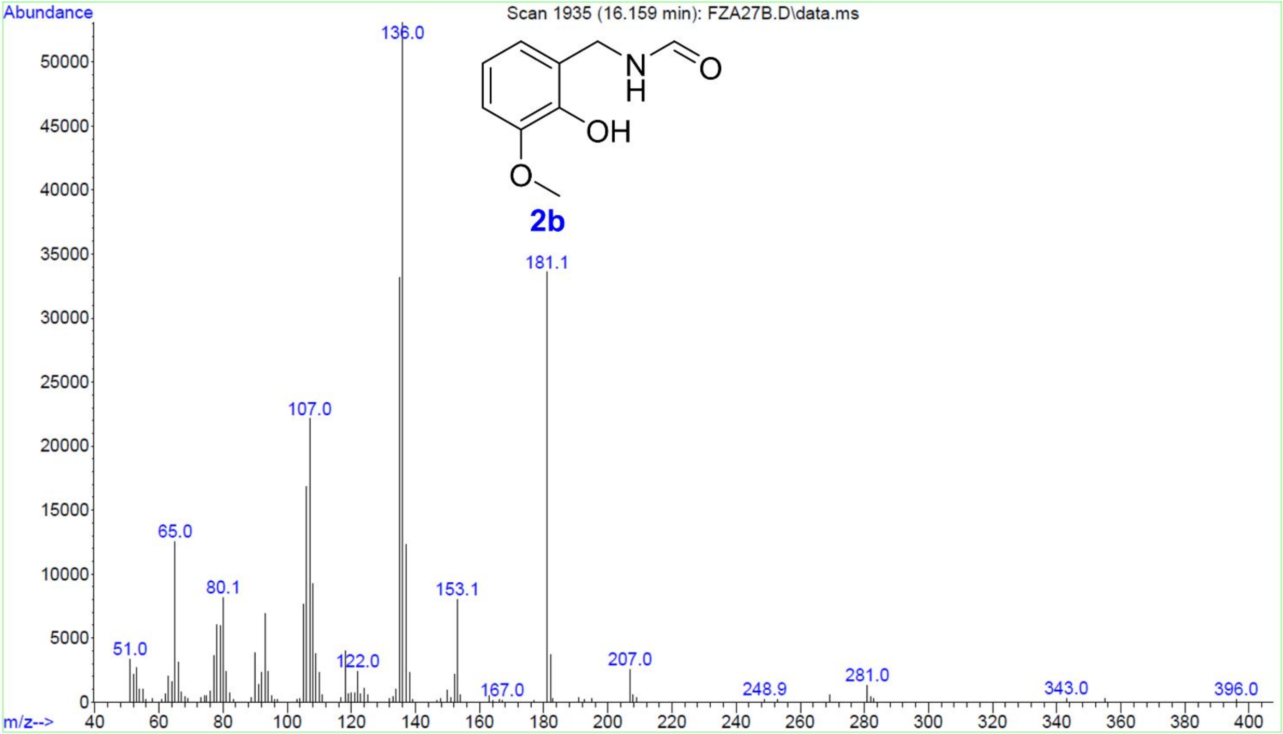

Figure S8: MS-spectrum of **2b** (m/z=181) obtained in LC-MS.

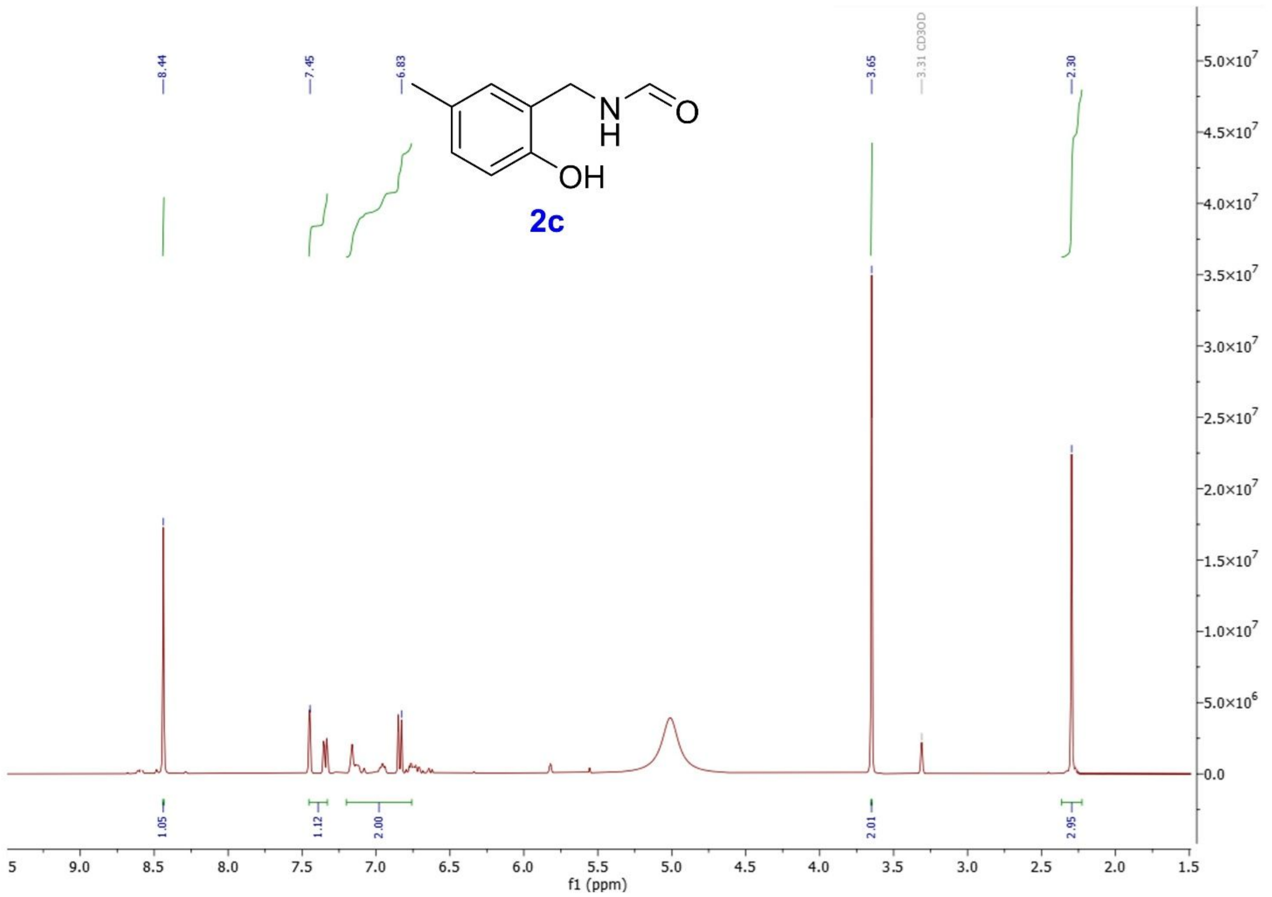

Figure S9: ^{1}H-NMR spectrum of **2c** [N-(2-hydroxy-5-methylbenzyl)formamide] (400 MHz, 298 K, CD_3OD-d4), δ, ppm: 2.3 s (3H, OCH_3), 3.65 s (2H, NCH_2), 6.83-7.45 m (3H, H_{arom}), 7.45 s (1H, OCH).

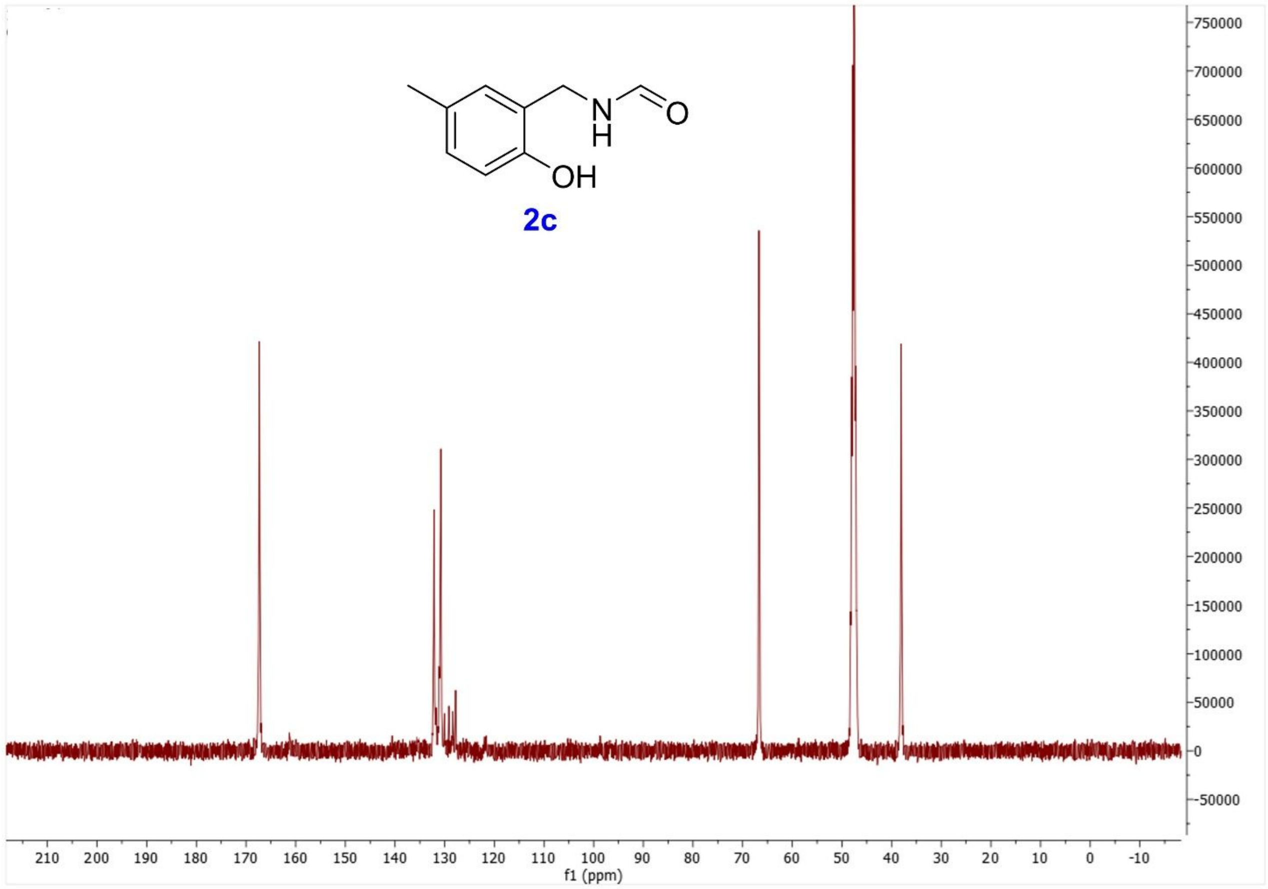

Figure S10: ^{13}C-NMR spectrum of **2c** (400 MHz, 298 K, CD_3OD-d4), δ, ppm 168.7 (C, -NHCHO), 132.0 (C_{arom}), 130.5 (C_{arom}), 129.9 (C_{arom}), 129.3 (C_{arom}), 128.5 (C_{arom}), 128.0 (C_{arom}), 66.9 (C, NCH_2), 38.4.ppm (C, CH_3).

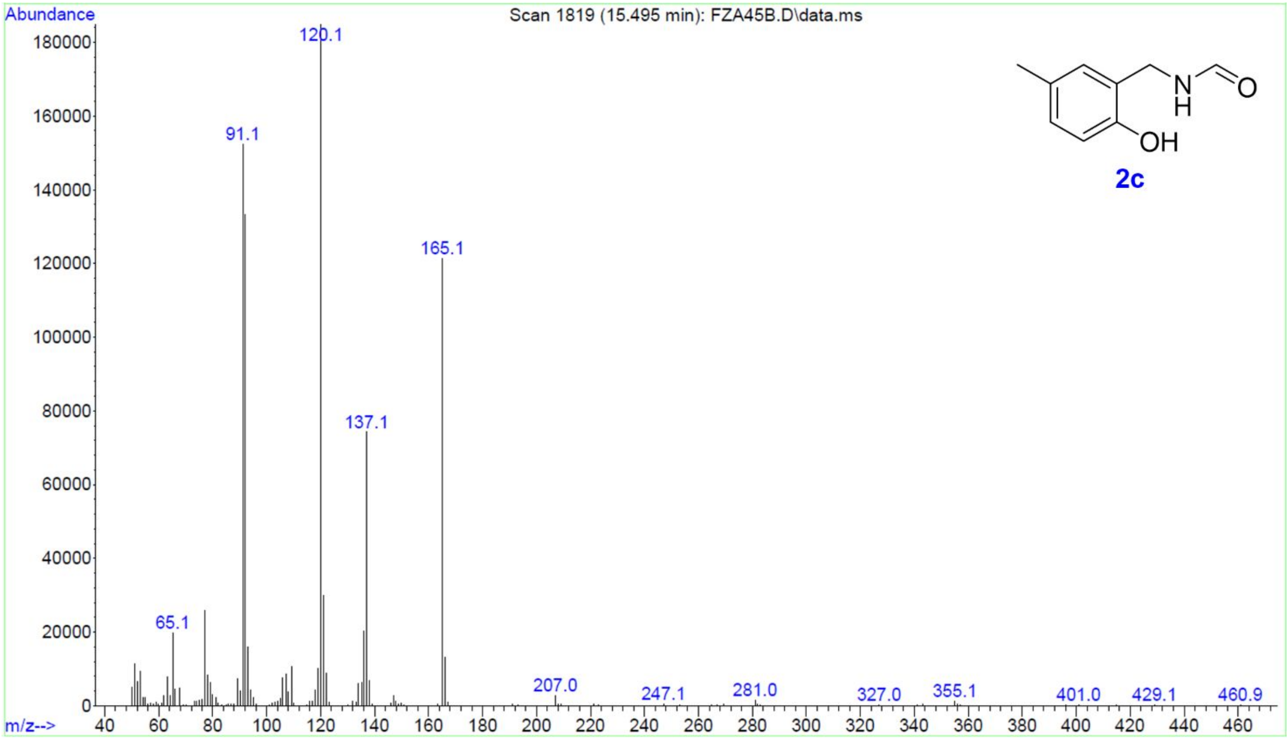

Figure S11: MS-spectrum of **2c** (m/z=165) obtained in GC-MS.

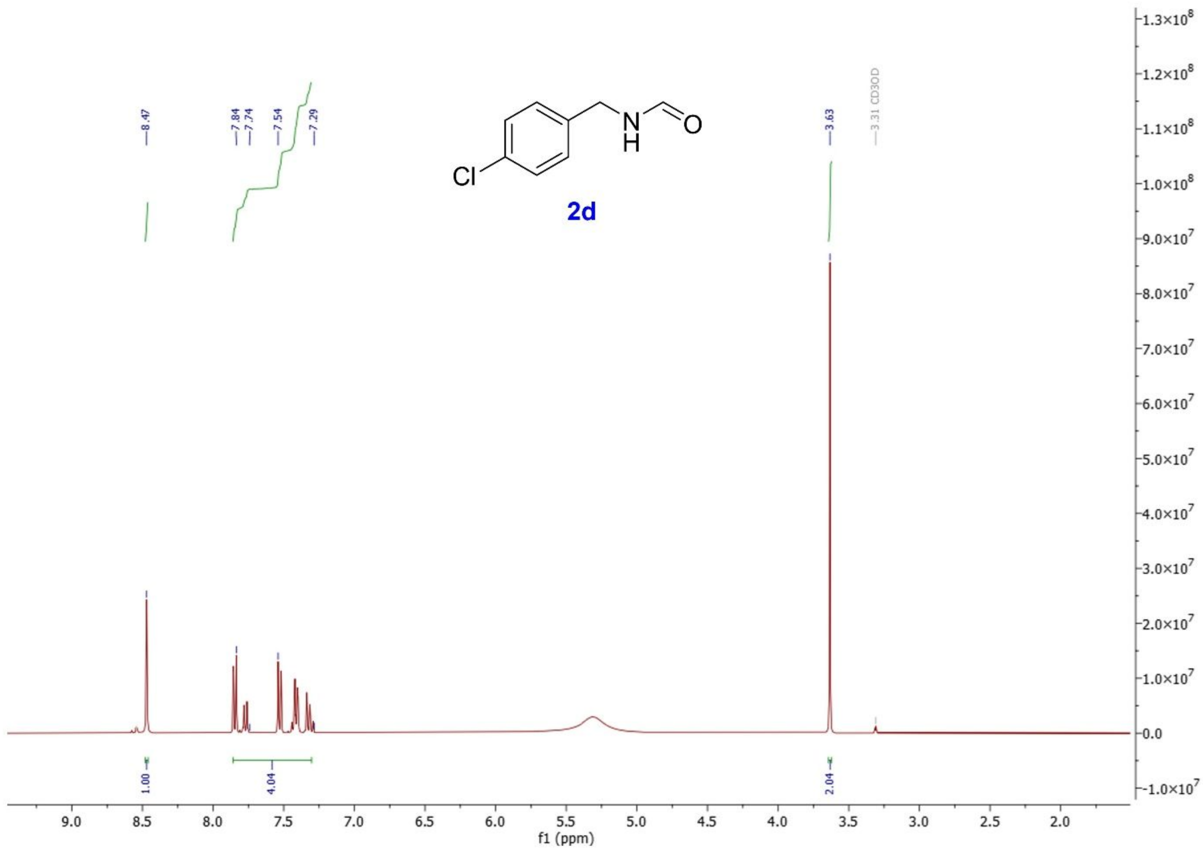

Figure S12: ^{1}H-NMR spectrum of **2d** [N-(4-chlorobenzyl)formamide] (400 MHz, 298 K, CD_3OD-d4), δ, ppm: 3.63 s (2H, NCH_2), 7.54-7.84 m (4H, H_{arom}), 8.47 s (1H, OCH).

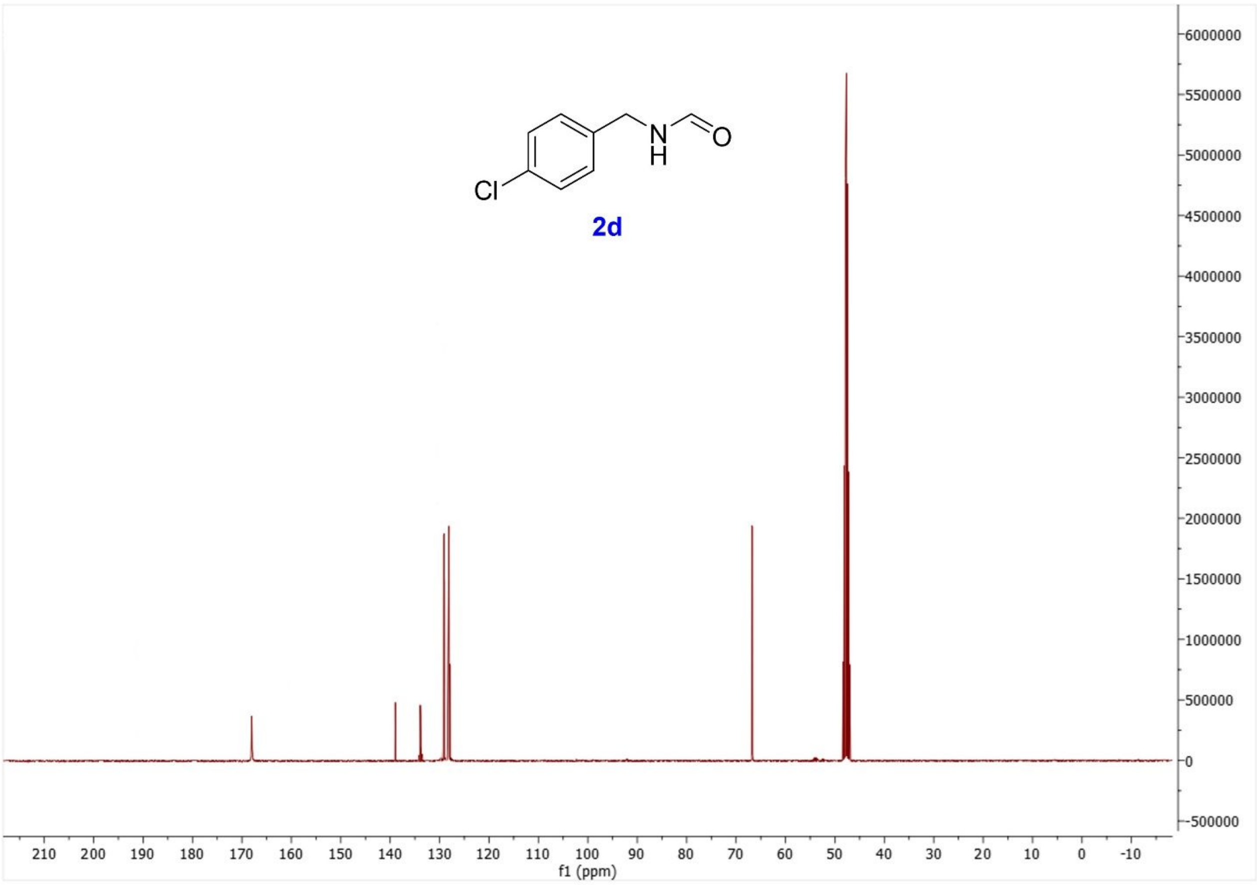

Figure S13: ^{13}C-NMR spectrum of **2d** (400 MHz, 298 K, CD_3OD-d4), δ, ppm 169.5 (C, -NHCHO), 139.0 (C_{arom}), 134.5 (C_{arom}), 129.9 (C_{arom}), 128.5 (C_{arom}), 67.2 (C, NCH_2).
The NMR characterization of product **2d** has also been previously reported in the literature.[3]

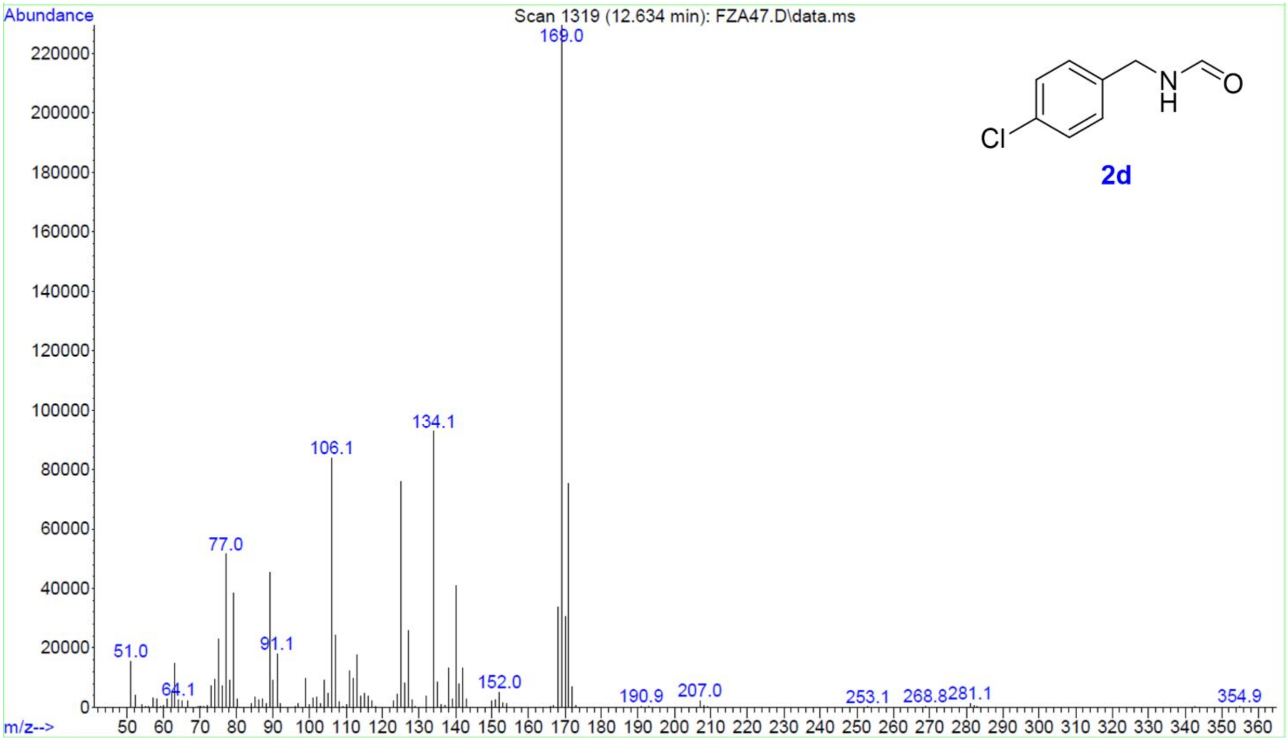

Figure S14: MS-spectrum of **2d** (m/z=169) obtained in GC-MS.

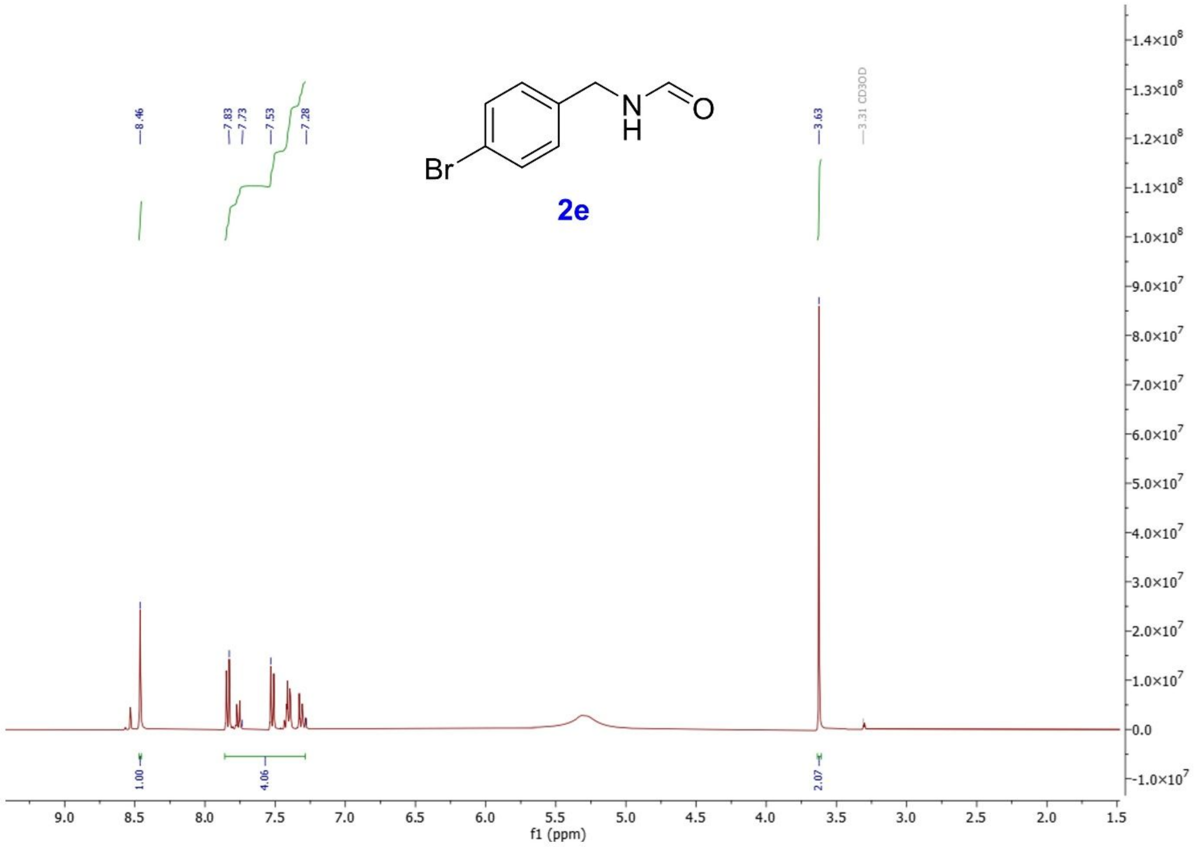

Figure S15: ^{1}H-NMR spectrum of **2e** [N-(4-bromobenzyl)formamide] (400 2-methoxy-4-(morpholinomethyl)phenolMHz, 298 K, CD_3OD-d4), δ, ppm: 3.63 s (2H, NCH_2), 7.53-7.83 m (4H, H_{arom}), 8.46 s (1H, OCH).

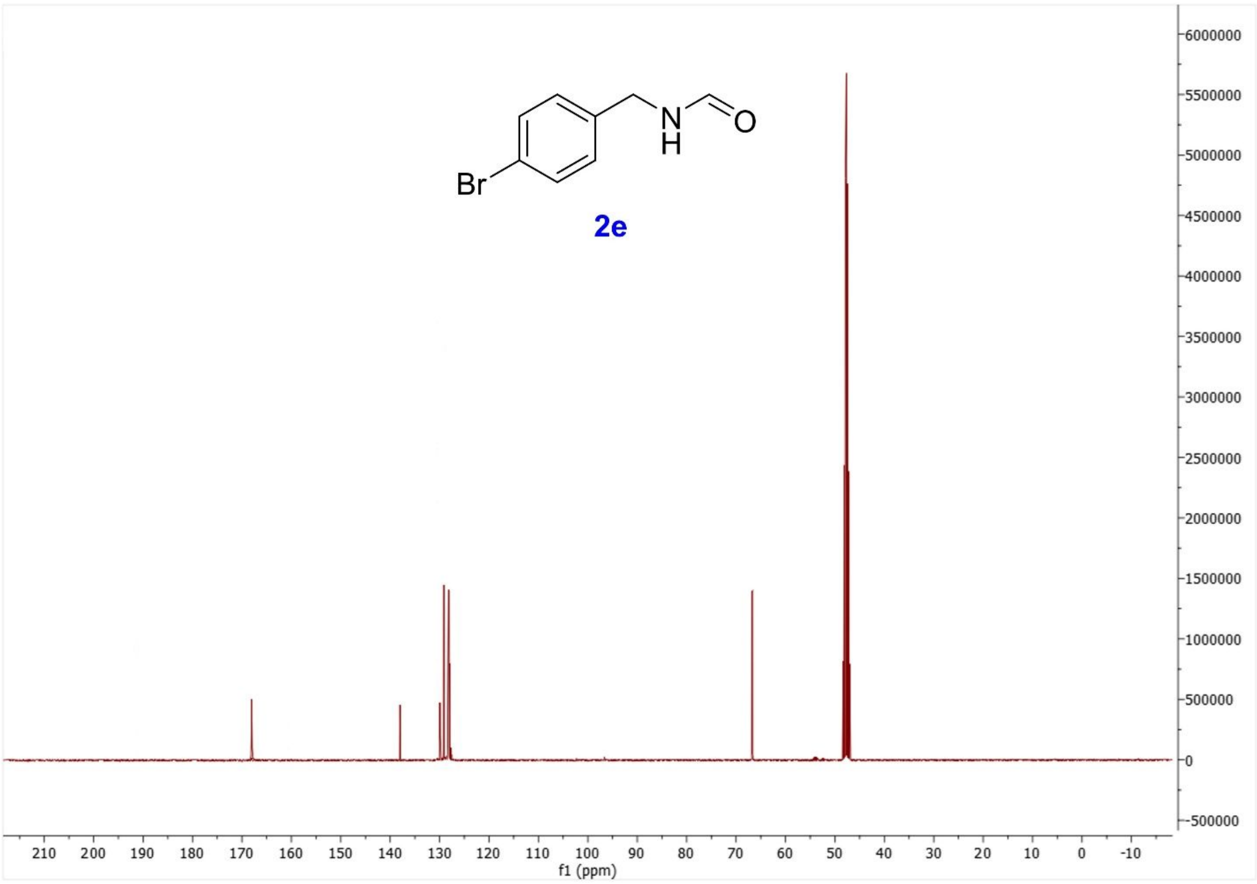

Figure S16: ^{13}C-NMR spectrum of **2e** (400 MHz, 298 K, CD_3OD-d4), δ, ppm 169.6 (C, -NHCHO), 138.0 (C_{arom}), 130.5 (C_{arom}), 129.9 (C_{arom}), 128.5 (C_{arom}), 67.5 (C, NCH_2).
The NMR characterization of product **2e** has also been previously reported in the literature.[3]

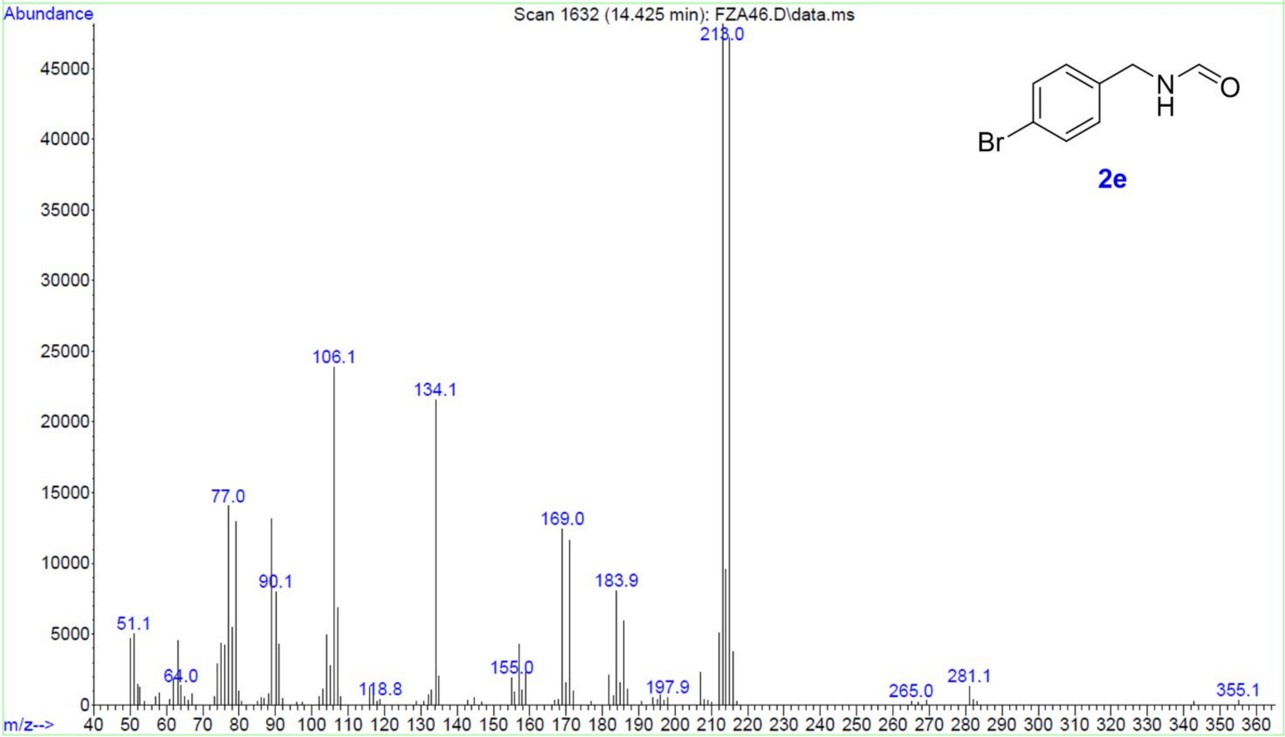

Figure S17: MS-spectrum of **2e** (m/z=213) obtained in GC-MS.

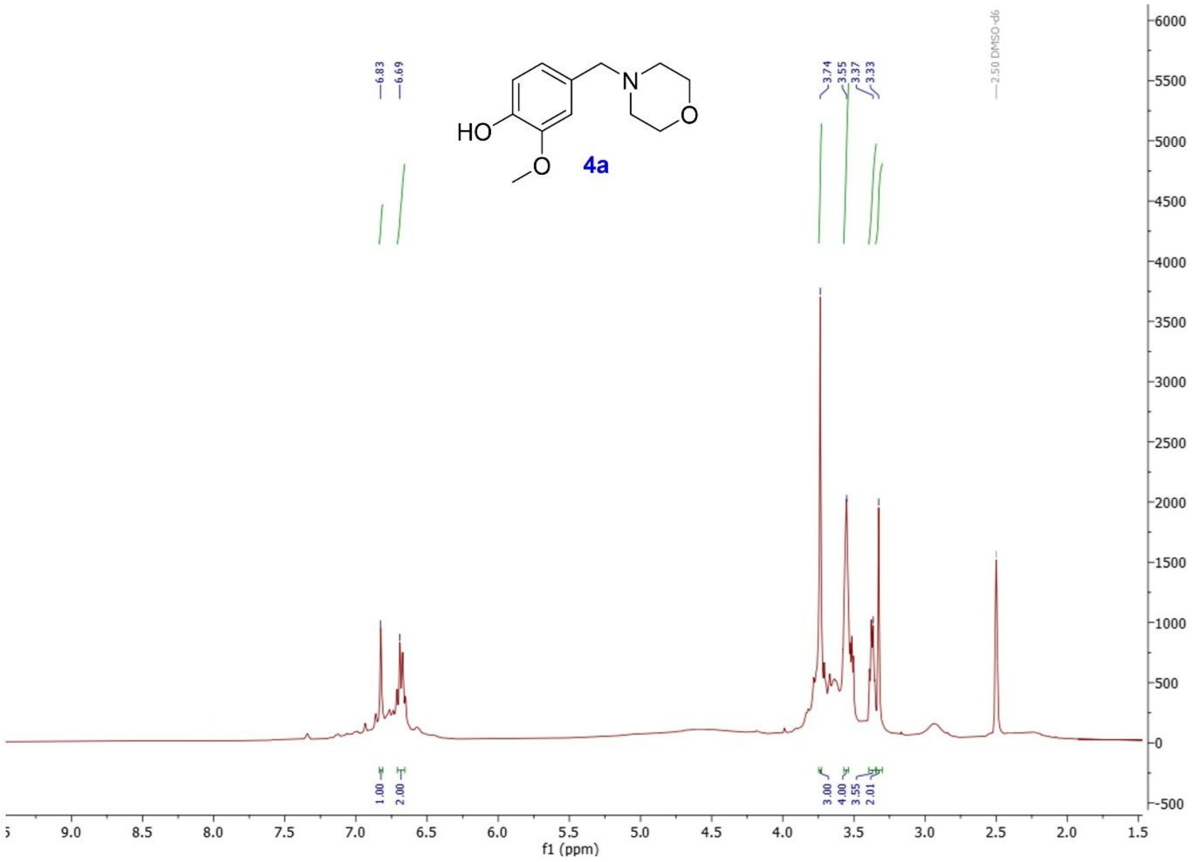

Figure S18: ^{1}H-NMR spectrum of **4a** [2-methoxy-4-(morpholinomethyl)phenol] (400 MHz, 298 K, $(CD_3)_2SO$-d6), δ, ppm: 3.33 s (2H, NCH_2), 3.37 m (4H, $2CH_{2\ morph}$), 3.55 m (4H, $2CH_{2\ morph}$), 3.74 s (3H, OCH_3), 6.69-6.83 m (3H, H_{arom}).

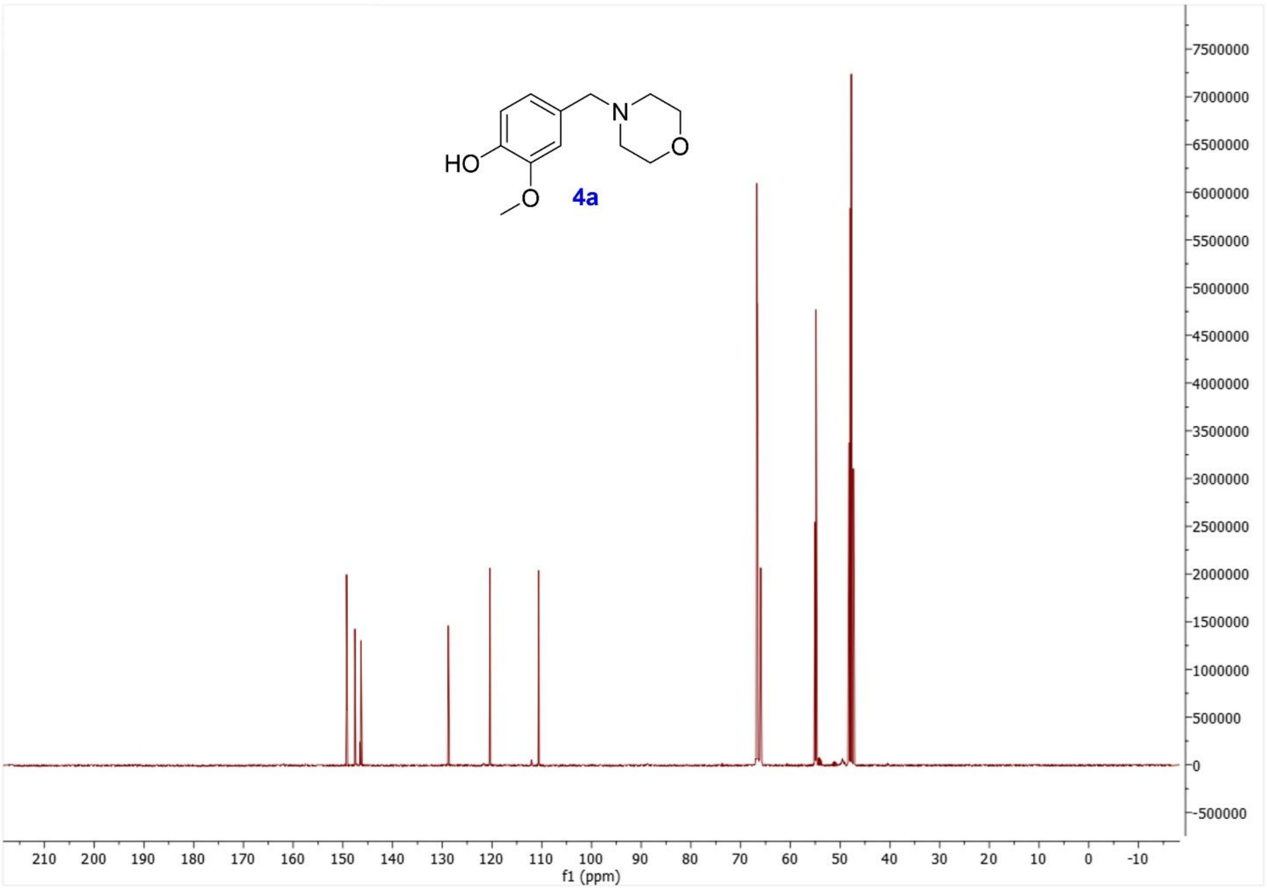

Figure S19: ^{13}C-NMR spectrum of **4a** (400 MHz, 298 K, CD_3OD-d4), δ, ppm, 149.0 (C_{arom}), 148.1 (C_{arom}), 147.3 (C_{arom}), 129.9 (C_{arom}), 120.0 (C_{arom}), 110.2 (C_{arom}), 66.4 (C, $-NCH_2$), 66.0 (C, $-NCH_2$), 55.1 (C, $-OCH_3$), 55.0 (C, $-OCH_2$).

The NMR characterization of product **4a** has also been previously reported in the literature.[4]

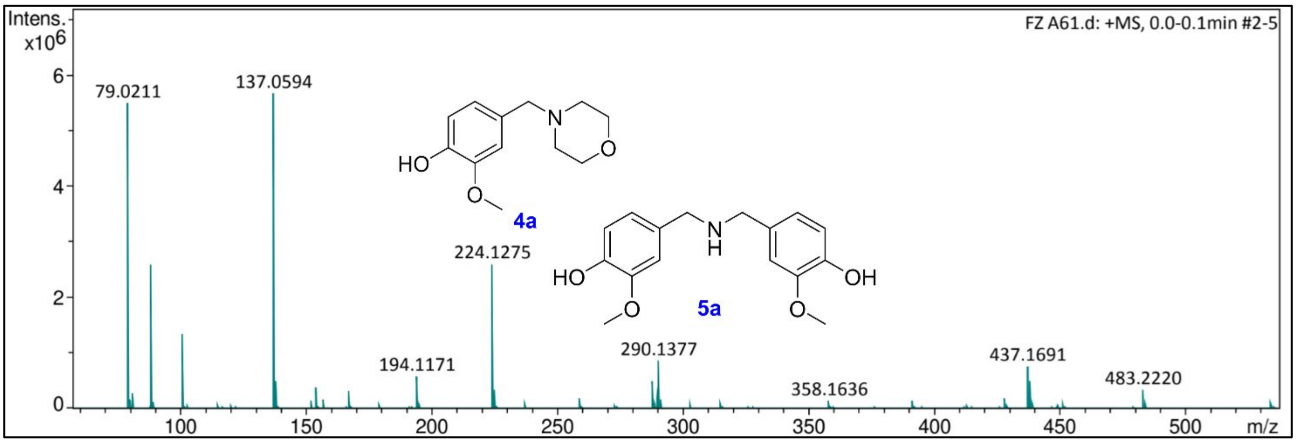

Figure S20: MS-spectra (LC-MS) of **4a** (m/z=224) and **5a** (m/z=290).

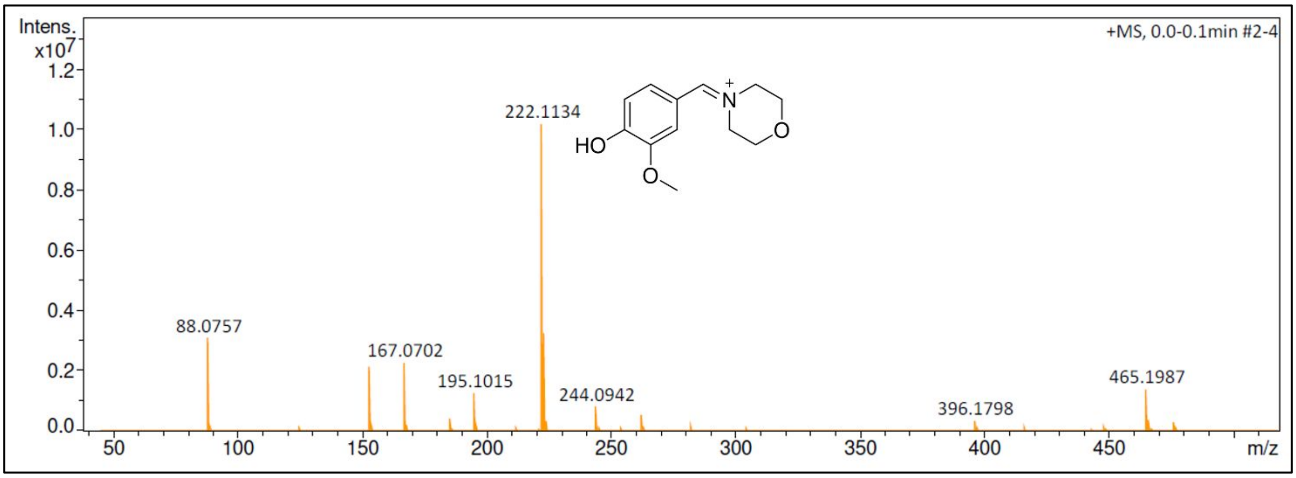

Figure S21: MS-spectrum of the **intermediate iminium ion 4a’** (m/z=222) obtained by LC-MS.

On the formation of 4-(4-hydroxy-3-methoxybenzylidene)morpholin-4-ium, an iminium species. At 100 °C and 50 rpm, under reactive extrusion conditions, the reaction of vanillin and morpholine (5 mmol each), and ammonium formate (3 mol equivs, 15 mmol) provided mostly 4-(4-hydroxy-3-methoxybenzylidene)morpholin-4-ium (Fig. S11) even after 60 minutes. According to the general mechanism of formation of iminium ions, these species generate from the condensation of a sec-amine and a carbonyl after a protonation process and water elimination. In the reaction studied in this work, the most likely source of protons was HCOOH derived from the thermal dissociation of ammonium formate.

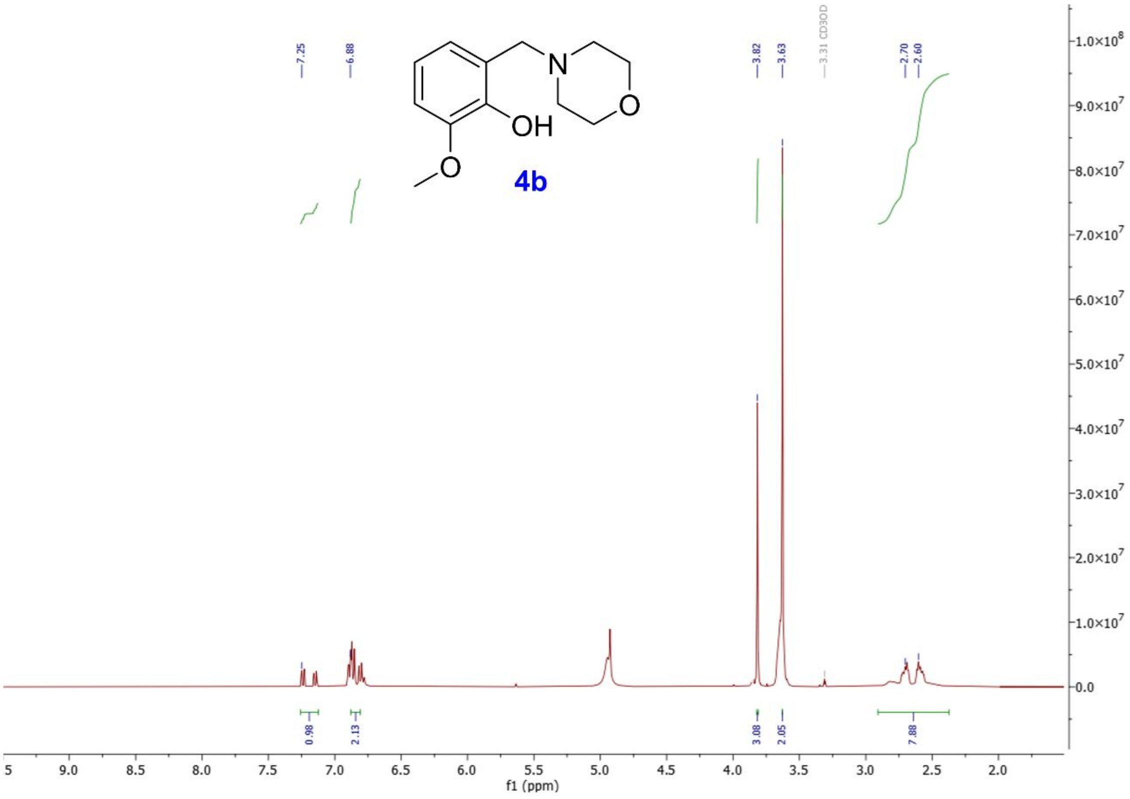

Figure S22: ^{1}H-NMR spectrum of **4b** [2-methoxy-6-(morpholinomethyl)phenol] (400 MHz, 298 K, CD_3OD-d4), δ, ppm: 2.60-2.70 m (8H, $4CH_{2\ morph}$), 3.63 s (2H, NCH_2), 3.82 s (3H, OCH_3), 6.88-7.25 m (3H, H_{arom}).

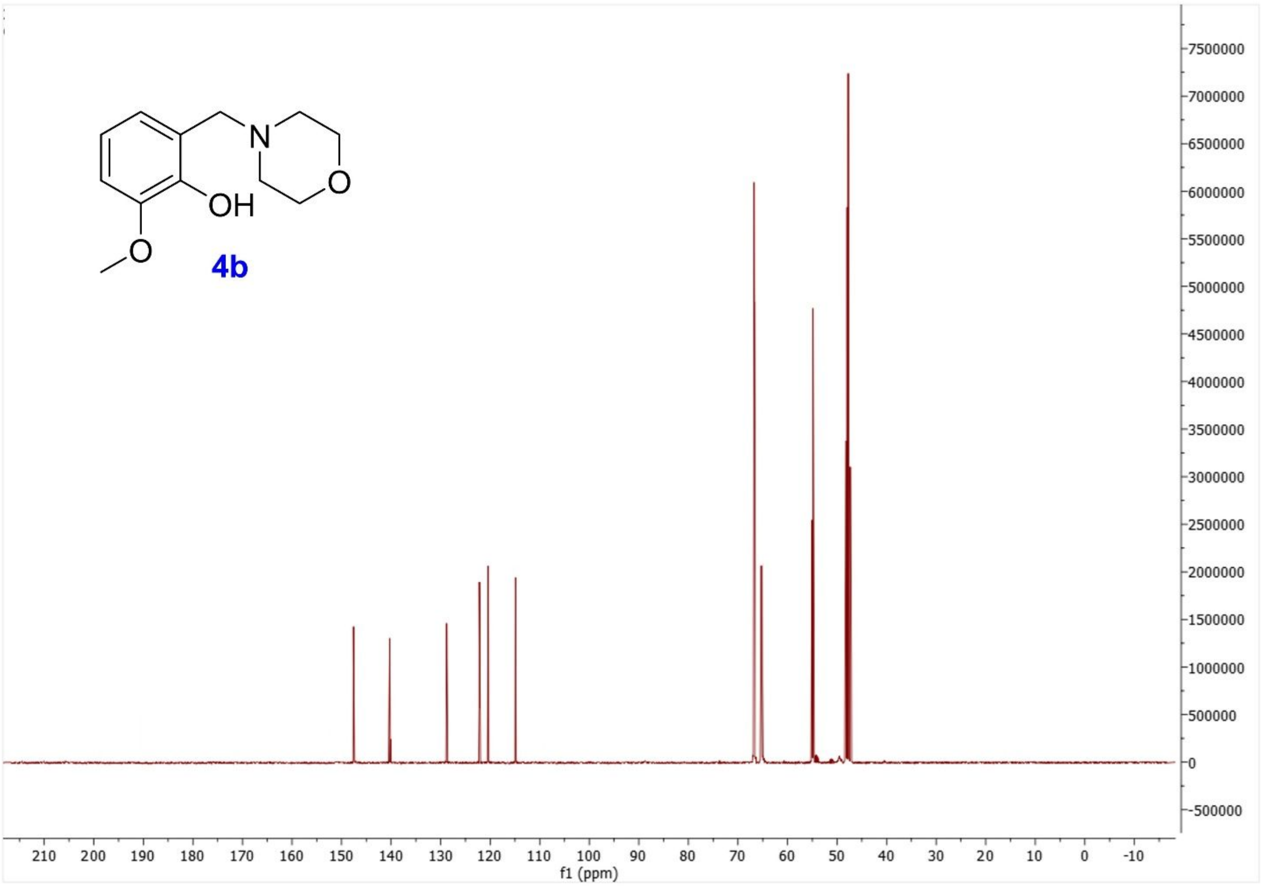

Figure S23: ^{13}C-NMR spectrum of **4b** (400 MHz, 298 K, CD_3OD-d4), δ, ppm, 148.5 (C_{arom}), 140.1 (C_{arom}), 129.3 (C_{arom}), 122.2 (C_{arom}), 121.0 (C_{arom}), 114.9 (C_{arom}), 66.5 (C, -NCH_2), 66.1 (C, -NCH_2), 55.1 (C, -OCH_3), 55.0 (C, -OCH_2).

The NMR characterization of product **4b** has also been previously reported in the literature.[5]

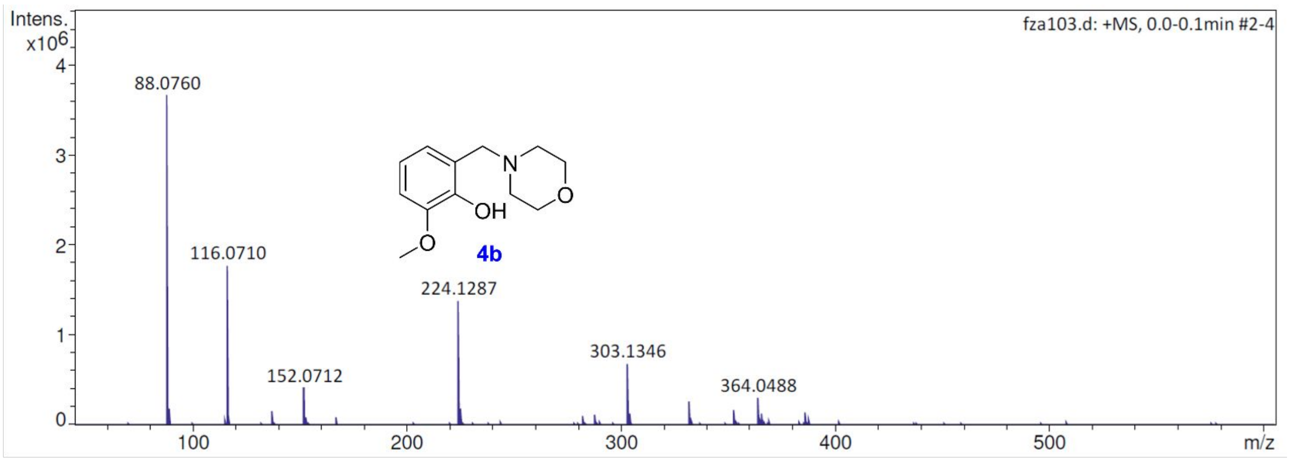

Figure S24: MS-spectrum of **4b** (m/z=224) obtained by LC-MS.

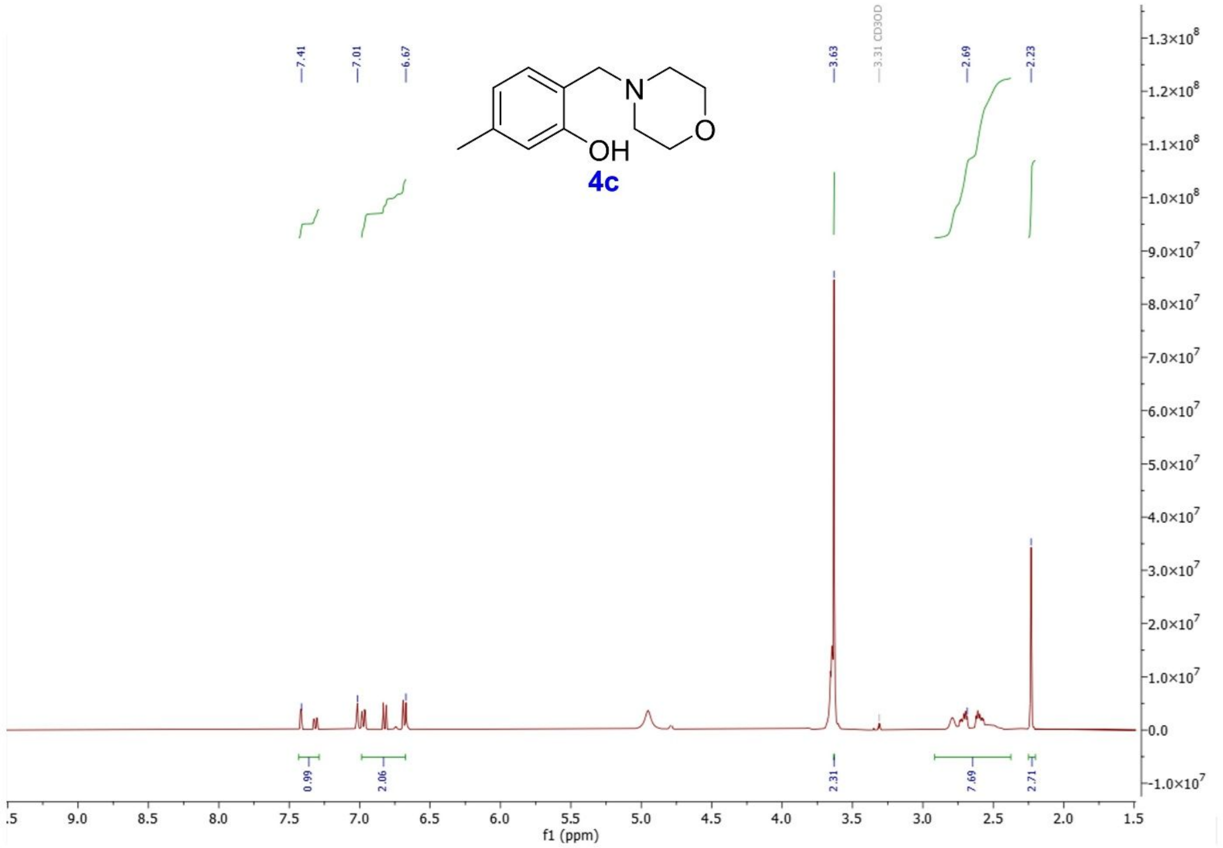

Figure S25: ^{1}H-NMR spectrum of **4c** [5-methyl-2-(morpholinomethyl)phenol] (400 MHz, 298 K CD_3OD-d4), δ, ppm: 2.23 s (3H, CH_3), 2.69-2.70 m (8H, $4CH_{2\,morph}$), 3.63 s (2H, NCH_2), 6.67-7.41 m (3H, H_{arom}).

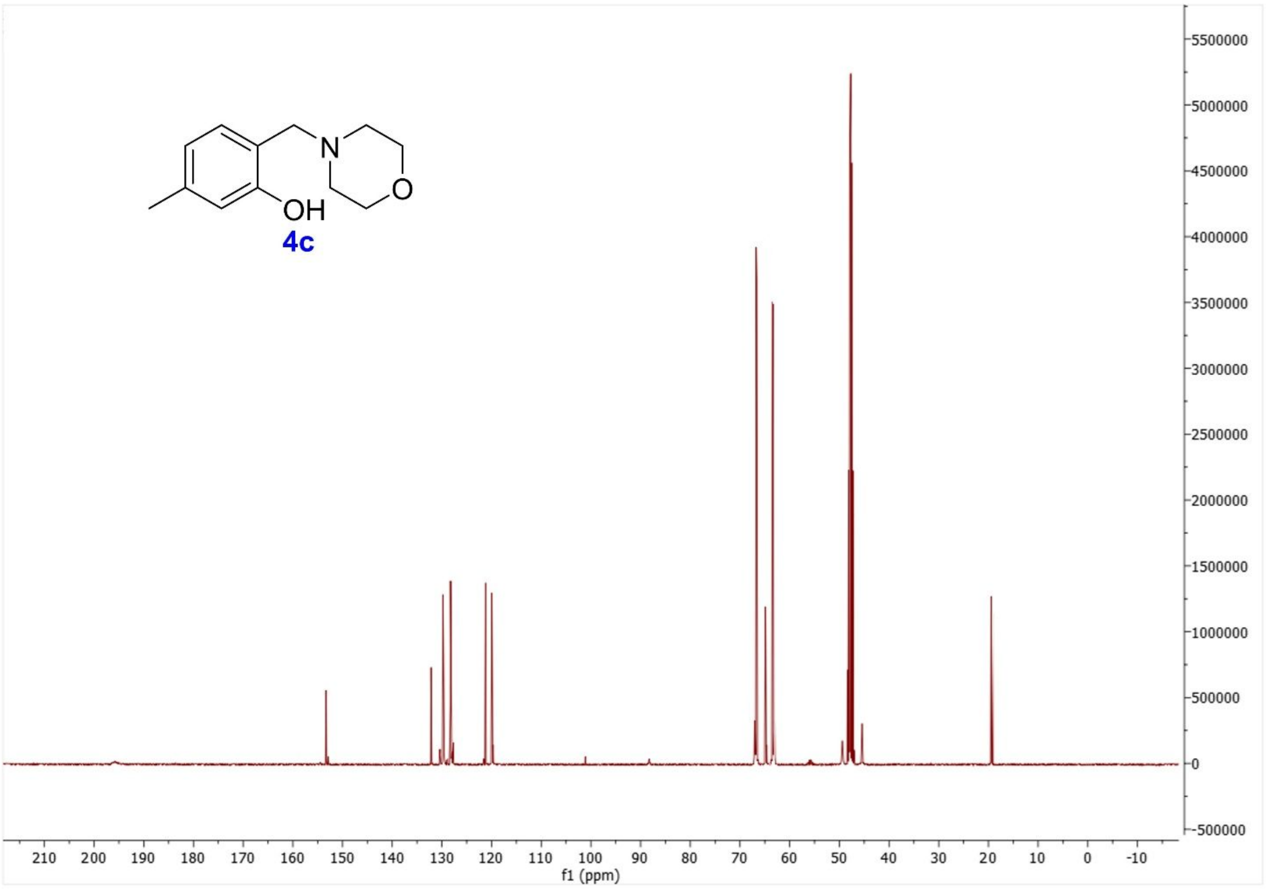

Figure S26: ^{13}C-NMR spectrum of 4c (400 MHz, 298 K, CD_3OD-d4), δ, ppm, 154.8 (C_{arom}), 133.1 (C_{arom}), 130.0 (C_{arom}), 129.4 (C_{arom}), 121.0 (C_{arom}), 120.2 (C_{arom}), 66.5 (C, -NCH_2), 65.2 (C, -NCH_2), 64.0 (C, -OCH_2), 20.0.ppm (C, CH_3).

The NMR characterization of product **4c** has also been previously reported in the literature.[6]

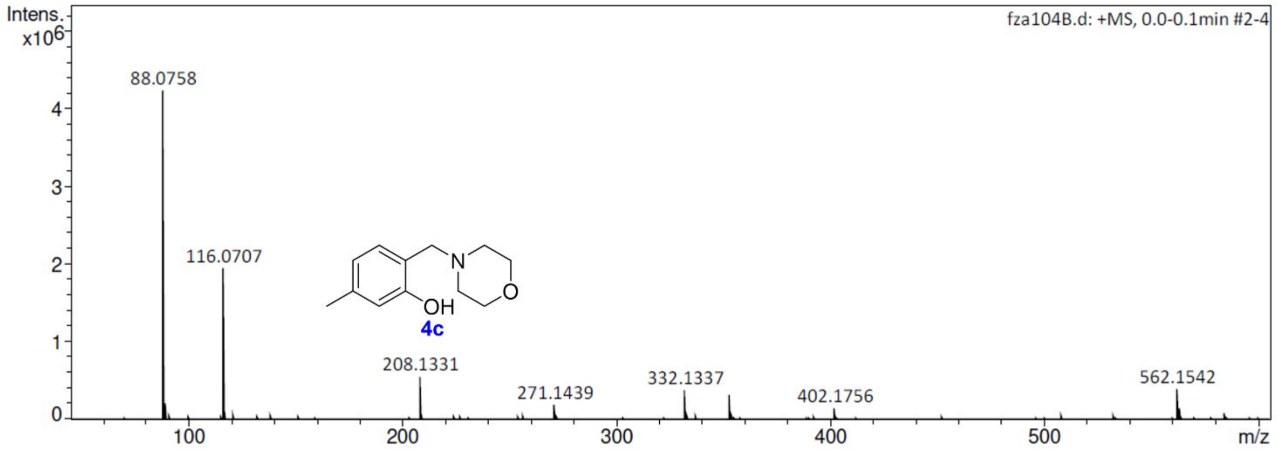

Figure S27: MS-spectrum of **4c** (m/z=208) obtained in LC-MS.

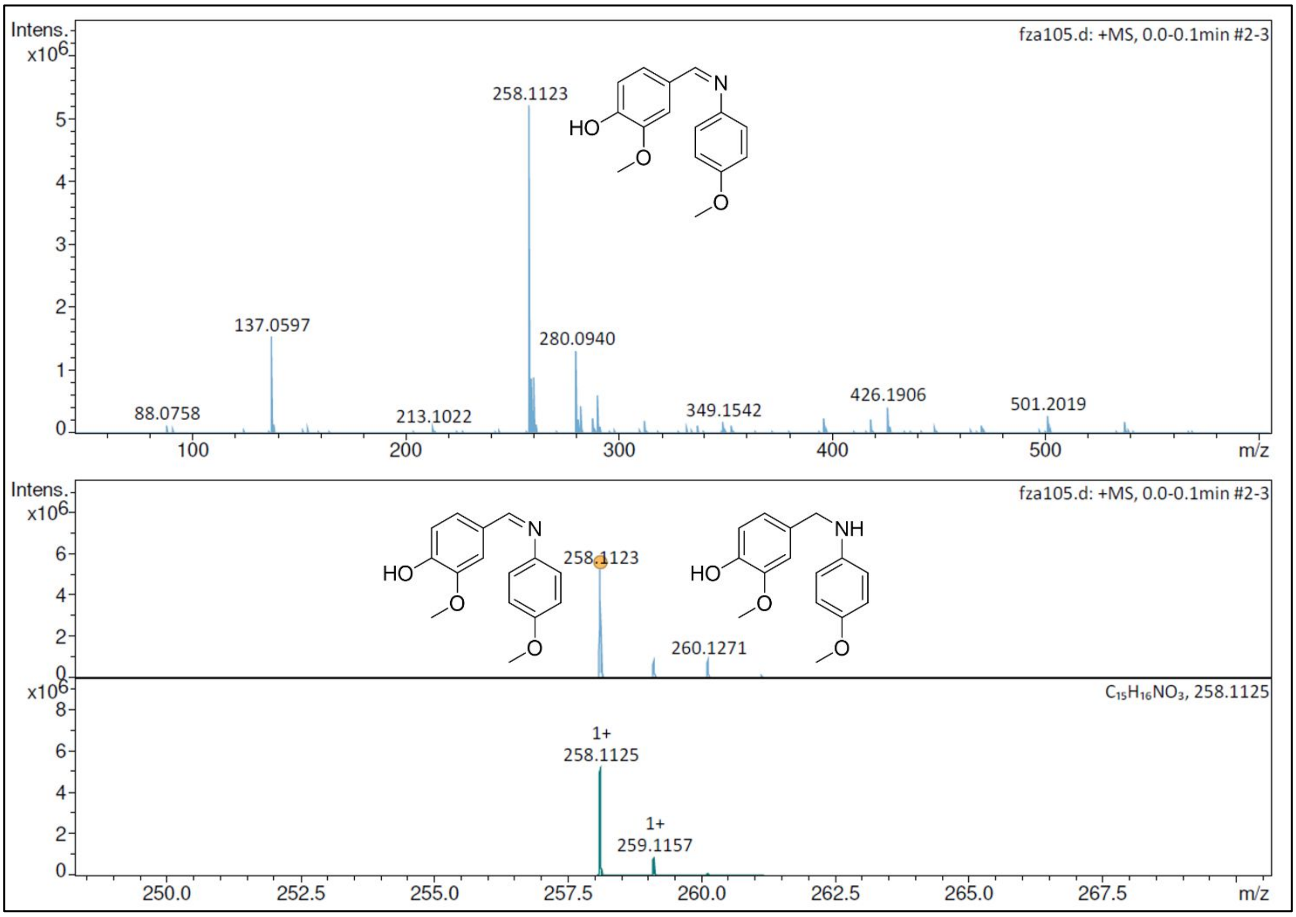

Figure S28: MS-spectra (LC-MS) of the imine formed from vanillin and anisidine (m/z=258), and traces of the corresponding amine (m/z=259).

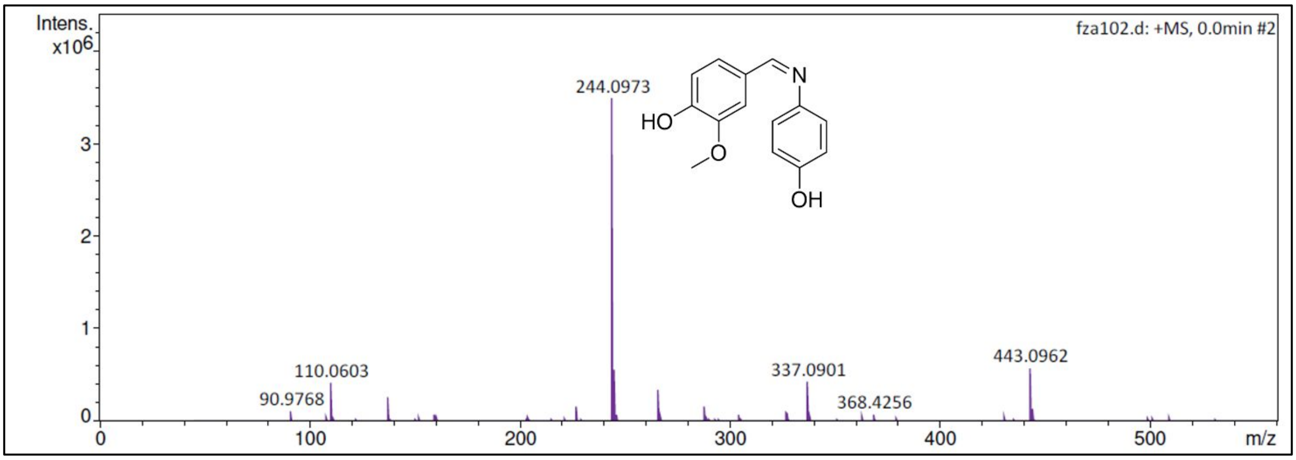

Figure S29: MS-spectrum (LC-MS) of the imine formed from vanillin and 4-aminophenol (m/z=244). No traces of the corresponding amine were detected.

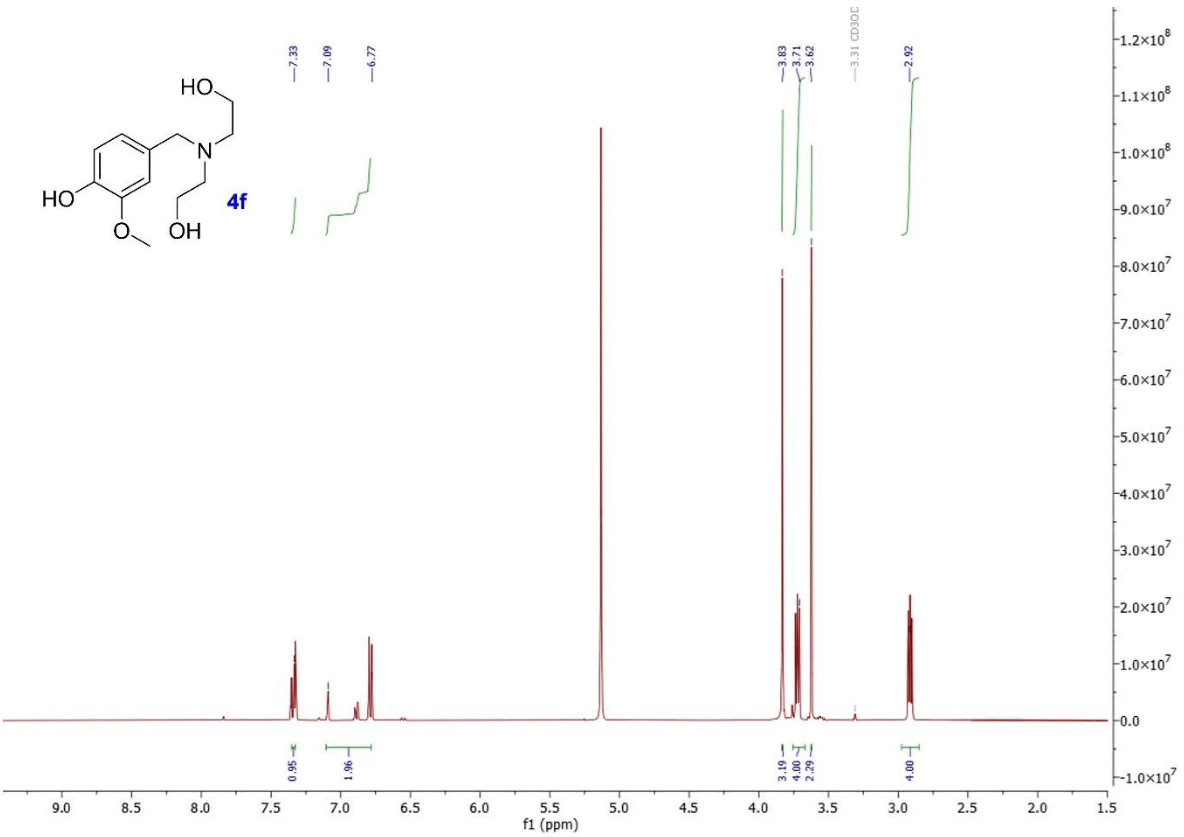

Figure S30: ^{1}H-NMR spectrum of **4f** (400 MHz, 298 K, CD3OD-d4), δ, ppm: 2.92 t (4H, $2CH_{2\ diethanolamine}$), 3.62 s (2H, NCH_2), 3.71 t (4H, $2CH_2$), 3.83 s (3H, OCH_3), 6.77-7.09 m (3H, H_{arom}), 7.33 s (1H, OH).

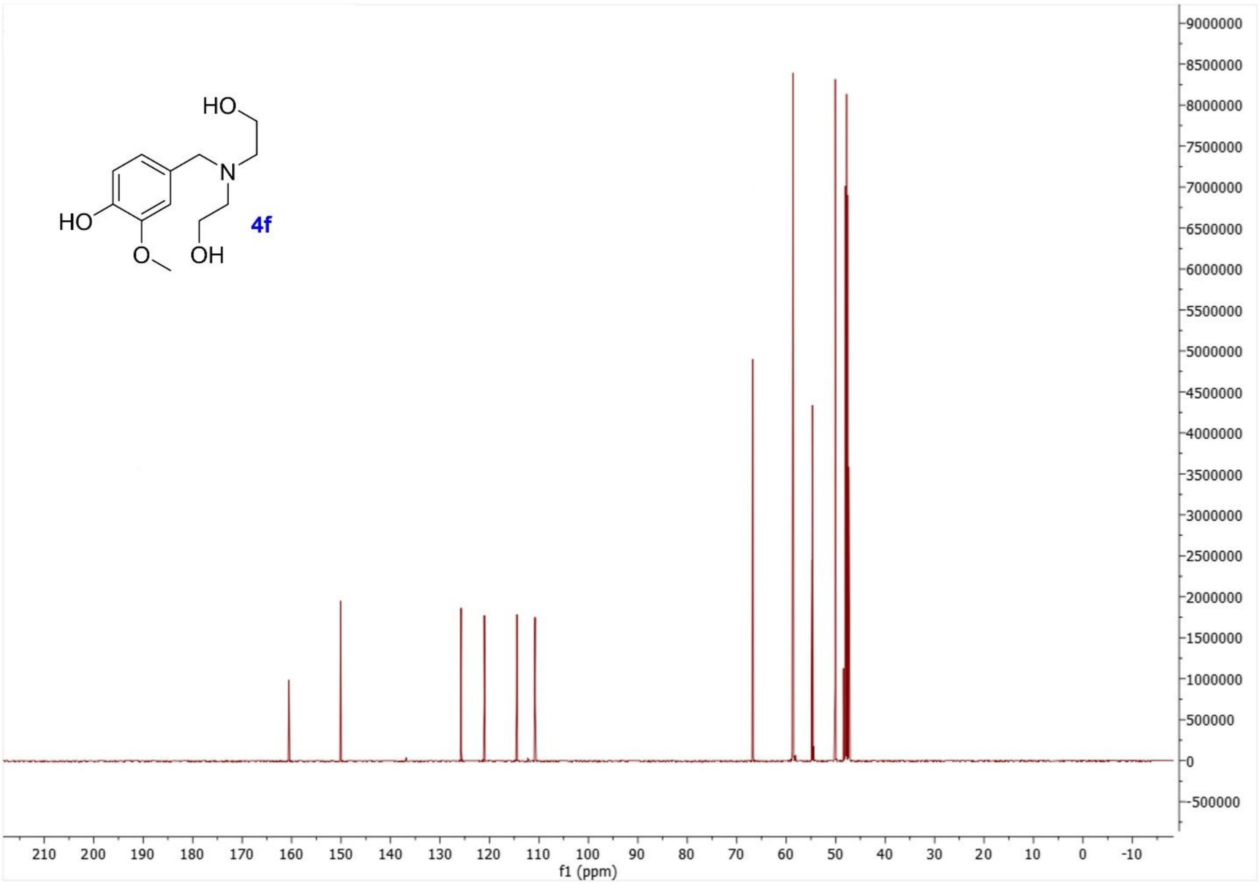

Figure S31: ^{13}C-NMR spectrum of **4f** [2,2'-((4-hydroxy-3-methoxybenzyl)azanediyl)bis(ethan-1-ol)] (400 MHz, 298 K, CD_3OD-d4), δ, ppm, 161.2 (C_{arom}), 150.1 (C_{arom}), 125.2 (C_{arom}), 121.4 (C_{arom}), 115.0 (C_{arom}), 110.4 (C_{arom}), 66.8 (C, -NCH_2), 59.2 (C, -NCH_2), 55.0 (C, -OCH_2), 50.0.ppm (C, O-CH_3). Additional characterization data for product **4d** have also been previously reported in the literature.[7]

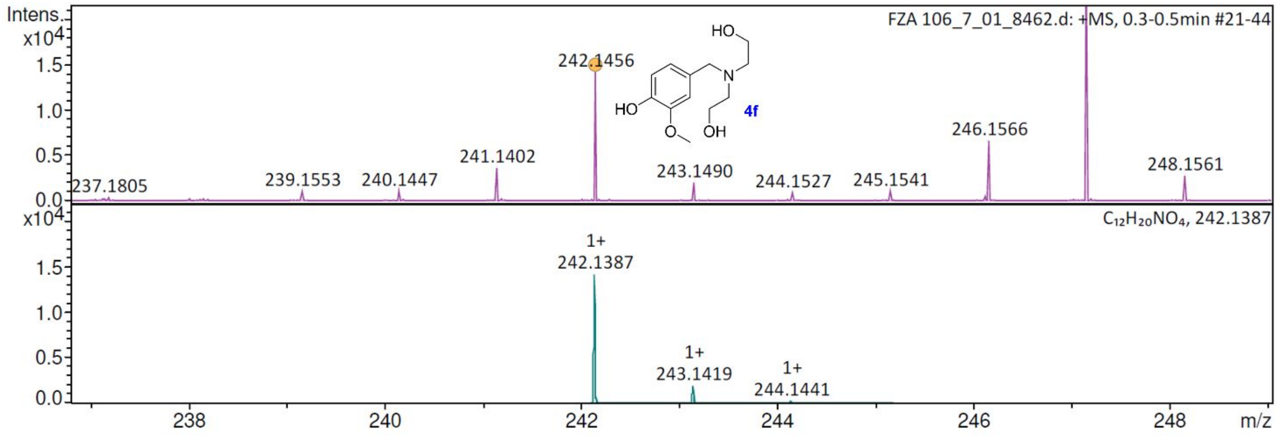

Figure S32: MS-spectrum of **4f** (m/z=241) obtained in LC-MS.

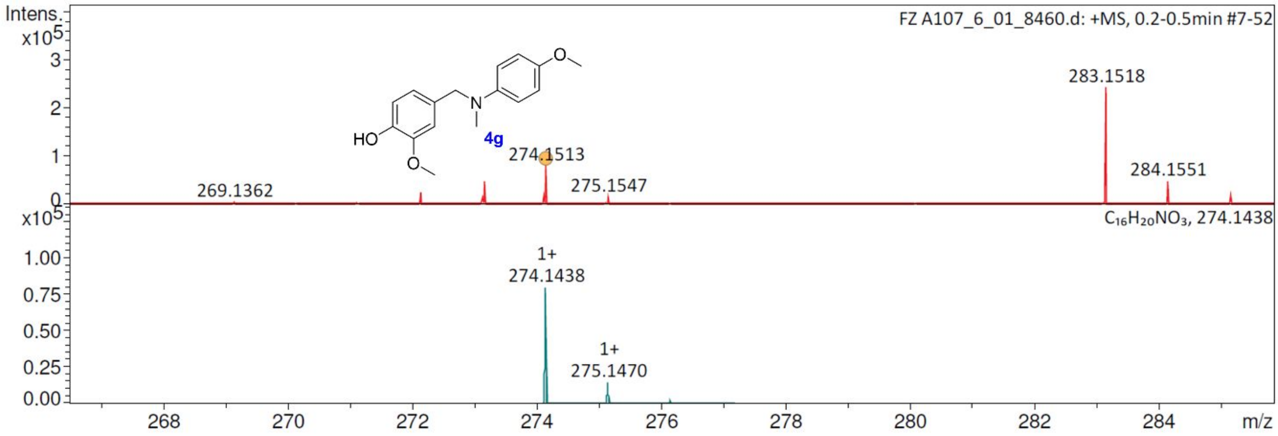

Figure S33: MS-spectrum of **4g** (m/z=273) obtained in LC-MS.

Characterization data for an analogue of product **4g** have also been reported previously in the literature.[8]

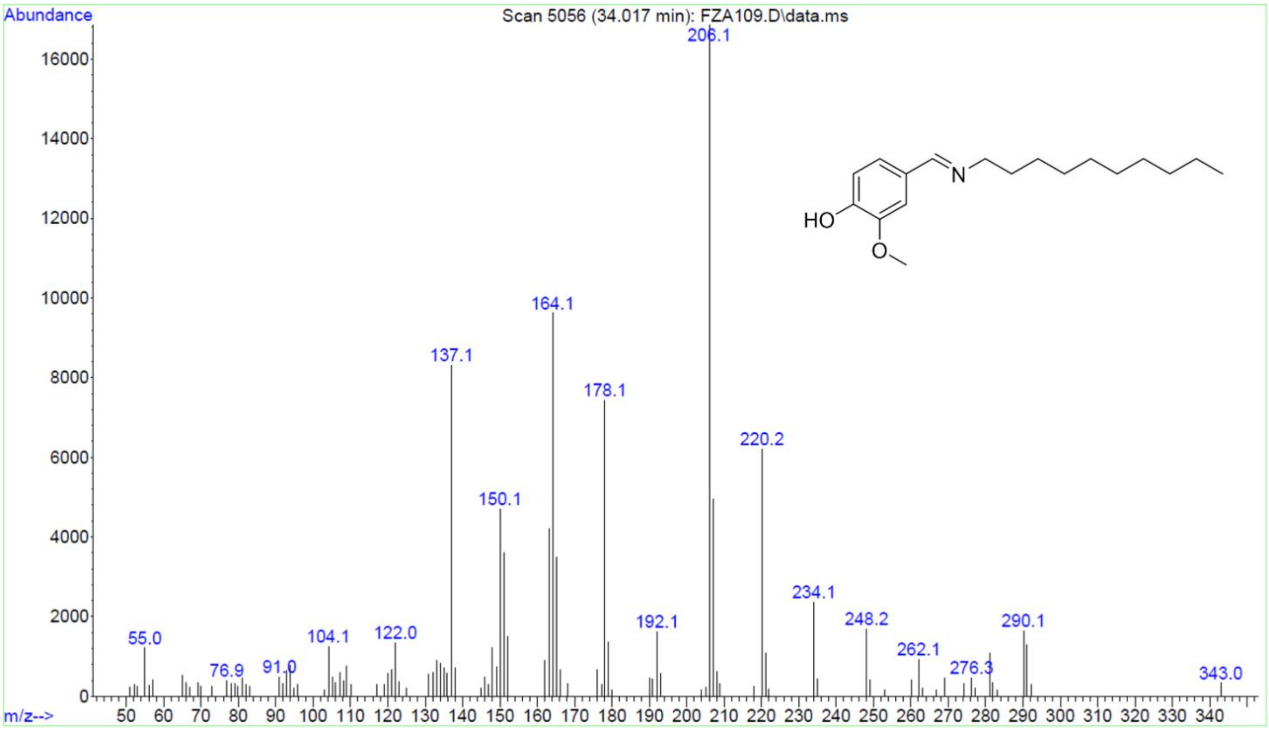

Figure S34: MS-spectrum (GC-MS) of the imine formed from vanillin and decylamine (m/z=291). No traces of the corresponding amine were detected.

Green Metrics Evaluation for the synthesis of amines

Conditions of entries 2, 5 and 6 of Table 2 were considered to compare the metrics of the synthesis of amine **4a** via the reactive extrusion. The CHEM21 toolkit's Zero Pass assessment was used. Results are reported in Figure S17. The increase of the Q ratio from 2 to 3 had minimal effects on conversion and selectivity which were almost steady (C=0.99 and S=0.81-0.84). These values earned a green (preferred) and an amber (acceptable with some issues flag, respectively, according to the CHEM21 criteria. Even the Optimum Efficiency (OE=0.75-0.77) did not change significantly with Q. The most notable impact was on RME that decreased from 0.47 to 0.39 as a consequence of an increased generation of byproducts (ammonia, carbon dioxide and water), and lower efficiency on the resource use. The E-factor reported in Table S1 was calculated considering the formation of water as a waste during the reaction. Overall, this analysis illustrates the trade-offs between reducing reagent excess and optimizing reaction efficiency.

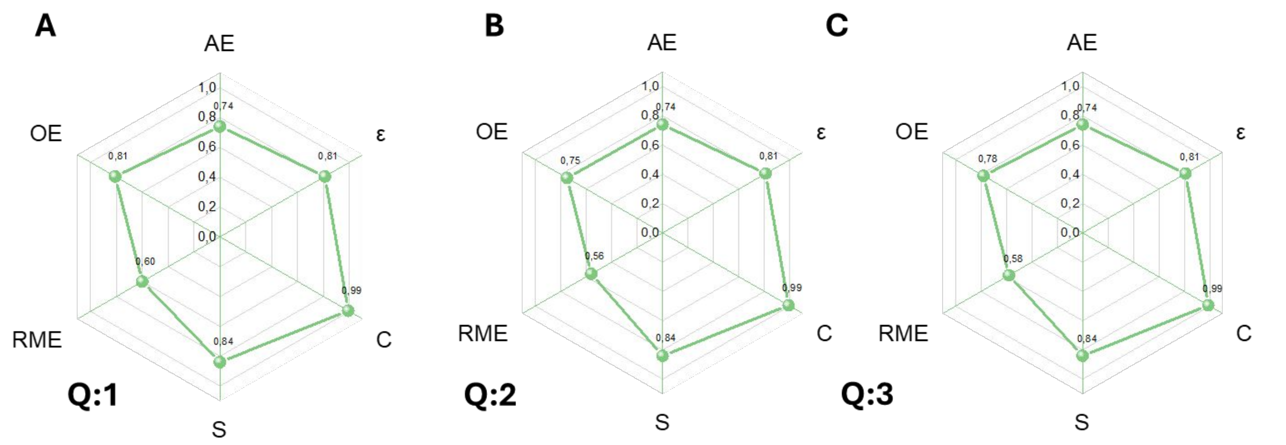

Figure S35. Hexagon radial chart for the evaluation of the metrics of the synthesis of amine **4a** via the extrusion-assisted reaction of vanillin, morpholine, and ammonium formate. Conditions of Table 2: A: entry 5, B: entry 6, C: entry 2.

Table S1. E-factor (E) and Process Mass Intensity (PMI) of the described processes involving: i) the Extrusion-based Leuckart reaction between vanillin and ammonium formate and ii) The Leuckart-type reductive amination involving vanillin, ammonium formate and morpholine. Q stands for equivalents of ammonium formate in respect to vanillin.

**Process**	**Q**	**E-factor**	**PMI**
Leuckart reaction	**3**	0.88	1.88
Leuckart-type Reductive Amination	**1**	0.92	1.92
Leuckart-type Reductive Amination	**2**	1.02	2.02
Leuckart-type Reductive Amination	**3**	1.13	2,13

References

1 M. Serafini, A. Griglio, S. Aprile, F. Seiti, C. Travelli, F. Pattarino, G. Grosa, G. Sorba, A. A. Genazzani, S. Gonzalez-Rodriguez, L. Butron, I. Devesa, A. Fernandez-Carvajal, T. Pirali and A. Ferrer-Montiel, *J. Med. Chem.*, 2018, **61**, 4436–4455.

2 D. M. Musatov, E. V. Starodubtseva, O. V. Turova, D. V. Kurilov, M. G. Vinogradov, A. K. Rakishev and M. I. Struchkova, *Russ J Org Chem*, 2010, **46**, 1021–1028.

3 W. Huang, Q. Mei, S. Xu, B. An, M. He, J. Li, Y. Chen, X. Han, T. Luo, L. Guo, J. Hurd, D. Lee, E. Tillotson, S. J. Haigh, A. Walton, S. J. Day, L. S. Natrajan, M. Schröder and S. Yang, *Chemistry – A European Journal*, 2024, **30**, e202303289.

4 M. Amaral, H. Asiki, C. E. Sear, S. Singh, P. Pieper, M. M. Haugland, E. A. Anderson and A. G. Tempone, *RSC Med. Chem.*, 2023, **14**, 1344–1350.

5 J.-L. Dai, N.-Q. Shao, J. Zhang, R.-P. Jia and D.-H. Wang, *J. Am. Chem. Soc.*, 2017, **139**, 12390–12393.

6 J. Liu and G. Yuan, *Tetrahedron Letters*, 2017, **58**, 1470–1473.

7 D.-D. Li, X.-M. Zhao, N. Gu, S. Zhi and Z.-W. Tao, *Journal of Coordination Chemistry*.

8 J. R. Bernardo, S. C. A. Sousa, P. R. Florindo, M. Wolff, B. Machura and A. C. Fernandes, *Tetrahedron*, 2013, **69**, 9145–9154.
